# A *TIMM17A* Regulatory Network Contributing to Breast Cancer

**DOI:** 10.3389/fgene.2021.658154

**Published:** 2021-08-05

**Authors:** Jiajia Cai, Jianyun Chen, Ling Huang, Changxi Wang, Weiyun Zhang, Quan Zhou, Zhaohui Sun

**Affiliations:** ^1^Department of Laboratory Medicine, General Hospital of Southern Theatre Command of PLA, Guangzhou, China; ^2^The First College of Clinical Medical, South Medical University, Guangzhou, China; ^3^IT and Cloud Computing Center, Geneplus-Shenzhen, Shenzhen, China

**Keywords:** *TIMM17A*, breast cancer, data mining, regulatory network, cell cycle, CDK1

## Abstract

**Background:**

Translocase of inner mitochondrial membrane 17A (*TIMM17A*) is overexpressed in breast cancer (BRCA), and upregulation can increase the aggressiveness of BRCA cells. This study examined the influence of the *TIMM17A* gene network on BRCA outcome.

**Methods:**

Expression levels of *TIMM17A* were compared between normal and tumor tissues from the Oncomine^TM^ database, and the association with patient survival was analyzed using Kaplan–Meier Plotter. Clinical factors influencing *TIMM17A* expression were studied by UALCAN. cBioPotal was then used to identify genes interacting with *TIMM17A*, and network relationships were assessed using the R clusterProfiler package. The association between *TIMM17A* mutation and mRNA expression in BRCA was examined using the LinkFinder application in LinkedOmics, and coexpressed genes were assessed for functional enrichment using the LinkInterpreter application. Furthermore, *TIMM17A* expression correlation with cell cycle phase distribution was performed by flow cytometry. Finally, the target networks of kinases, microRNAs (miRNAs), and transcription factors were identified using GeneMANIA. The expression and correlation of potential miRNAs and targets were further validated in BRCA cell lines by qRT-PCR.

**Results:**

Expression of *TIMM17A* was significantly elevated in BRCA compared with normal tissue (*p* < 0.05), and overexpression was associated with both poor overall survival (OS) and shorter distant metastasis-free survival (DMFS) (*p* < 0.05). Expression of *TIMM17A* was not associated with age, sex, BRCA subclass, clinical stage, or patient ethnicity. The coexpressed *TIMM17A* network was enriched in genes targeted by cell cycle regulators such as *CDK1*, *miR-331*, and *E2F* family transcription factors (*FDR* < 0.001). Furthermore, flow cytometry revealed a strong association between higher *TIMM17A* expression and faster cell cycle progression in these BRCA cell lines. In addition, expression of *TIMM17A* protein was correlated with CDK1 protein expression in BRCA cell lines as measured by western blotting.

**Conclusion:**

Elevated *TIMM17A* expression accelerates the progression of BRCA, thereby reducing OS and DMFS. The *TIMM17A*-associated networks identified here provide clues to the molecular pathogenesis of BRCA and potential targets for BRCA treatment.

## Introduction

According to recent global estimates, breast cancer (BRCA) is now the most frequent cause of cancer-related mortality among adult females (Bray et al., 2018). However, BRCA is a highly heterogeneous clinical entity, with multiple subtypes according to distinct histological features and treatment sensitivity profiles (Harbeck and Gnant, 2017; Yeo and Guan, 2017; Liang et al., 2020). This heterogeneity has also been confirmed at the gene expression level by high-throughput molecular profiling (Ahn et al., 2020; Krug et al., 2020). More precise delineation of these BRCA subtypes may eventually yield more efficacious individualized treatments (Zardavas et al., 2015). Currently, however, therapeutic options for many cancers are limited by our relatively poor understanding of the underlying molecular and cellular mechanisms (Garrido-Castro and Winer, 2018). Malignancy results from the disruption of multiple coordinated gene networks (Garrido-Castro and Winer, 2018), so elucidation of the gene network changes characterizing various BRCA subtypes may facilitate the design of patient-specific interventions (Garrido-Castro and Winer, 2018; Radvanyi, 2018; Zacharakis et al., 2018).

Translocase of inner mitochondrial membrane 17A (*TIMM17A*) is an essential component of the highly conserved TIM23 translocase complex involved in mitochondrial protein import and metabolism (Bomer et al., 1996). Expression of *TIMM17A* is elevated in BRCA (Kannangai et al., 2007; Yang et al., 2016). Furthermore, *TIMM17A* upregulation can increase the aggressiveness of BRCA cells (Yang et al., 2016) and is associated with poor clinical outcome in human BRCA patients (Salhab et al., 2012). Indeed, Xu at el. identified *TIMM17A* as a biomarker for poor outcome from BRCA (Xu et al., 2010; Salhab et al., 2012).

All genes operate within multiple networks of coregulated and functionally related genes. However, *TIMM17A* networks are not well described. In this study, we examined the associations between clinical outcome measures and *TIMM17A* expression levels among BRCA cases from The Cancer Genome Atlas (TCGA). Furthermore, we constructed a network of genes coregulated with *TIMM17A* in BRCA patients to reveal new potential molecular targets for diagnosis and treatment.

## Materials and Methods

### Statistical Analysis

Statistical analyses were performed using SPSS Software version 26.0 (IBM SPSS Statistics, Chicago, IL, United States) and GraphPad Prism 7 (GraphPad Software, San Diego, CA, United States). Overall survival (OS) was compared between BRCA patients with high or low *TIMM17A* expression by the Kaplan–Meier method and log-rank test (Mishra et al., 2019; Okano et al., 2019). The associations between *TIMM17A* expression levels and various clinicodemographic features were analyzed by Spearman’s correlations. Group means were compared by independent samples *t*-test (Chandrashekar et al., 2017). The association between *TIMM17A* mutation and mRNA expression in the TCGA BRCA cohort was assessed by *t*-test with correction for multiple comparisons (Vasaikar et al., 2018). *p*-Value less than 0.05 (two-tailed) was considered significant for all tests.

### Oncomine Analysis

Differences in *TIMM17A* expression and copy number between normal tissue and BRCA were evaluated for TCGA and Curtis datasets in the Oncomine database^1^.

### Kaplan–Meier Plotter Analysis

The association of tumor *TIMM17A* expression level with BRCA outcome was examined in total BRCA datasets, GSE45255, and GSE7390 using Kaplan–Meier Plotter (publicly available at https://kmplot.com/). For this analysis, we chose *TIMM17A* gene chip results (Affymetrix ID 215171_s_at) for estimation of mRNA expression level, both OS and distant metastasis-free survival (DMFS) as clinical outcome metrics, and a follow-up duration of 120 months.

### UALCAN Analysis

Clinical factors influencing *TIMM17A* expression were examined in the TCGA dataset using the ‘‘breast invasive carcinoma’’ section of UALCAN^2^. The influencing factors assessed were age, sex, tumor stage, BRCA subclass, and metastasis occurrence based on a previous factor analysis (Chandrashekar et al., 2017).

### c-BioPortal Analysis

Genes interacting with *TIMM17A* were identified in the “breast invasive carcinoma” dataset of Firehose Legacy using c-BioPortal^3^. Neighboring genes with alteration frequencies greater than 5% were selected for network analysis.

### clusterProfiler Analysis

The R 3.6.0 package cluster Profiler was used to visualize networks of genes related to functional changes identified by Disease Ontology (DO), Gene Ontology (GO), Kyoto Encyclopedia of Genes and Genomes (KEGG), and Reactome (Hayes, 2010). DO was used to identify genes associated with BRCA, GO to describe gene functions using standardized terminology, and KEGG to place genes into the following molecular interaction and reaction networks: metabolism, genetic information processing, environmental information processing, cellular processes, biological system, human diseases, and drug development. Reactome was used to supplement the results of KEGG analysis by visualizing various human reactions and biological channels. The Benjamini–Hochberg (BH) adjusted p method was used to determine significant enrichment at a cutoff of 0.05.

### LinkedOmics Analysis

The RNAseq dataset of the ‘‘breast invasive carcinoma’’ cohort was obtained from LinkedOmics^4^ (Vasaikar et al., 2018). The dataset including *TIMM17A* expression comprised 1,093 patients. LinkFinder was used to identify the association between *TIMM17A* mutation and mRNA expression in the TCGA BRCA cohort and Pearson’s correlation analysis to assess the strengths of the associations (positive and negative). Kinase and KEGG pathway analyses were then performed using the gene set enrichment analysis (GSEA) tool in LinkInterpreter.

### KEGG Pathway Analysis

KEGG is composed of 15 comprehensive database resources, a manually planned database, and four system information category databases generated by calculation. The KEGG pathway system can link gene expression to multiple “cells and biological functions” domains, including “metabolism,” “cellular processes,” “biological functions,” and “human diseases” (Kanehisa et al., 2017). We identified the pathways for 26 enriched neighboring genes by KEGG^5^.

### GeneMANIA Analysis

Protein--protein interaction (PPI) networks were constructed using GeneMANIA^6^ (Warde-Farley et al., 2010). For each network, we examined “physical interactions,” “coexpression,” predicted, “colocalization,” “pathway,” “genetic interactions,” and “shared protein domains.” The classified function were “protein targeting,” “mitochondrial transport,” “establishment of protein localization to mitochondrion,” “protein localization to mitochondrion,” “protein targeting to mitochondrion,” and (or) “mitochondrion organization.”

### Cell Culture

The human BRCA cell lines BT-549 and SK-BR-3 were obtained from the Shanghai Cell Bank Type Culture Collection Committee (Shanghai, China). Cells were cultured in complete growth medium as recommended by the distributor in a humidified incubator at 37°C under a 5% CO_2_ atmosphere.

### Cell Transfection

Expression of *TIMM17A* in cancer cell lines was manipulated by transfection with small interfering RNAs (siRNAs). A negative control siRNA (si-NC) and *TIMM17A*-targeting siRNA (si-TIMM17A) were designed and synthesized by LAIDEMENG (Guangzhou, China). Cell lines in logarithmic growth phase were seeded at 6 × 10^5^ cells/well in six-well plates, allowed to adhere overnight, and then incubated in transfection medium containing the indicated siRNA and Lipofectamine 3000 (Invitrogen, Waltham, MA, United States) according to the standard protocol of the manufacturer. After 6 h, the transfection medium was exchanged for complete medium and cells incubated for another 48 h to allow expression changes before subsequent experiments.

### Quantitative RT-PCR

Total RNA was extracted from cells using Trizol reagent (Invitrogen, CA, United States) according to the instructions of the manufacturer, and cDNAs generated using EvoM-MLV RT Premix. Gene expression levels were then measured by quantitative PCR on a Bio-Rad CFX RT-qPCR detection system using SYBR Premix Ex TaqII (Takara, Dalian, China). The 18S rRNA gene was used as an internal control. All samples were run in triplicate. Fold changes in gene expression were calculated using the ΔΔCt method. Details of the primer sequences used for qPCR are listed in Supplementary Table 8.

### Western Blotting

Whole cell extracts were prepared by incubation of harvested cells in ice-cold lysis buffer containing 1:100 PMSF and 1:100 protease inhibitor cocktail for 30 min. Lysates were then centrifuged at 14,000 rpm for 5 min at 4°C, and the supernatant was collected and stored at −80°C. Lysates were mixed with 5 × THE buffer at 4:1 (vol/vol), boiled for 10 min, cooled slowly to room temperature, centrifuged briefly, and stored at 20°C. Proteins were separated by sodium dodecyl sulfate polyacrylamide gel electrophoresis and electro-transferred (100 V at low temperature, 1 min/kDa) to polyvinylidene fluoride (PVDF) membranes pretreated with methanol for 5∼30 s, and transmembrane buffer for 20 min. Blotted membranes were then rinsed with Tris-buffered saline containing Triton-X (TBST) for 5 min, incubated in TBST with 5% skimmed milk powder rinsed in TBST for 8 min, and incubated in anti-TIMM17A, anti-CDK1, or anti-GAPDH (gel-loading control) at 4°C overnight. Immunoblotted membranes were then incubated with the indicated secondary antibody at 37°C for 50 min to 3 h, and washed three times in TBST (8 min/wash). Protein bands were visualized with chemiluminescence substrate (1∼5 min) and captured using MiniChemi imaging system (Bio-OI, Beijing, China).

### Flow Cytometry

The cell cycle progression of cancer cell lines was evaluated using a PI cell cycle Kit. Cells were harvested by trypsin digestion, washed twice in PBS, and fixed in 75% precooled ethanol for more than 2 h. Fixed cells were then treated with kit reagents according to the manufacturer’s protocol and analyzed using FACSCantoll flow cytometer (BD FACSCanto II, China).

## Results

### *TIMM17A* Expression in BRCA

**FIGURE 1:**
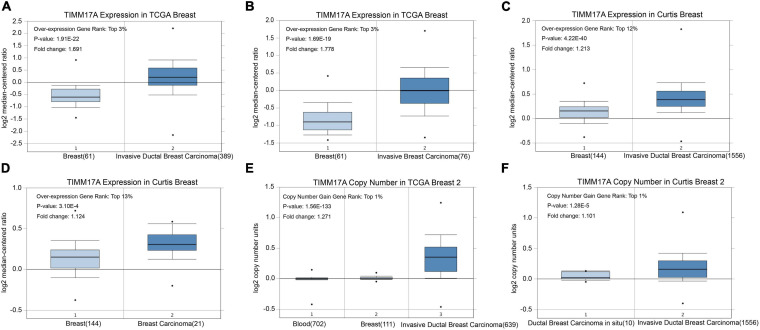
*TIMM17A* transcription in breast carcinoma (Oncomine^TM^). Levels of *TIMM17A* mRNA and DNA copy number were significantly higher in breast carcinoma than in normal tissue. Shown are fold change, associated *p* values, and overexpression rank based on Oncomine^TM^ analysis. **(A–D)** Box plots showing *TIMM17A* mRNA levels for The Cancer Genome Atlas (TCGA) Invasive Ductal Breast Carcinoma, TCGA Invasive Breast Carcinoma, Curtis Invasive Ductal Breast Carcinoma, and Curtis Breast Carcinoma datasets, respectively. **(E,F)** Box plots showing *TIMM17A* copy number in TCGA Breast 2 and Curtis Breast 2 datasets, respectively.

**FIGURE 2:**
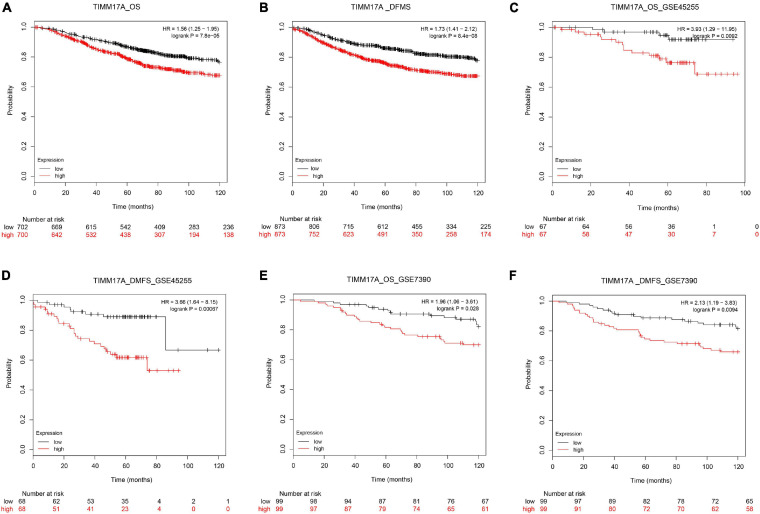
Kaplan–Meier survival curves showing significantly reduced overall survival (OS) and shorter distant metastases-free survival (DMFS) among breast cancer patients with high levels of *TIMM17A* expression. Clinical survival outcomes of breast cancer patients with high or low expression levels of *TIMM17A*. **(A)** OS in TCGA cohorts. **(B)** DMFS in TCGA cohorts. **(C)** OS in the GSE45255 cohort. **(D)** DMFS in the GSE45255 cohort. **(E)** OS in the GSE7390 cohort. **(F)** DMFS in the GSE7390 cohort. Plots are truncated at 10 years.

**FIGURE 3:**
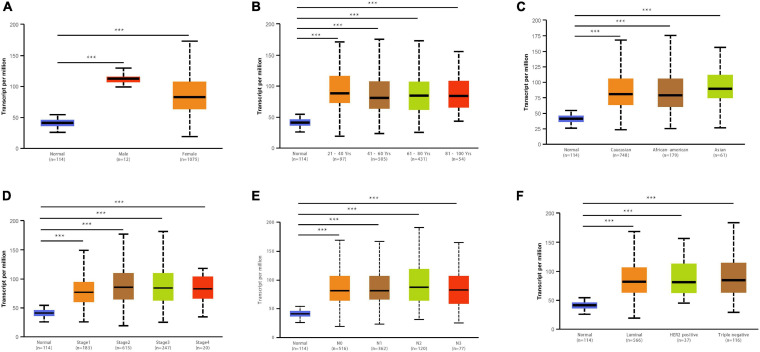
*TIMM17A* transcription in breast cancer (BRCA) patient subgroups stratified by sex, age, ethnicity, cancer stage, and metastasis (UALCAN). **(A)** Boxplot showing relative expression of *TIMM17A* in normal individuals of either sex and separately for male and female BRCA patients. **(B)** Boxplot showing relative expression of *TIMM17A* in normal individuals of any age and separately for BRCA patients aged 21–40, 41–60, 61–80, or 81–100 years. **(C)** Boxplot showing relative expression of *TIMM17A* in normal individuals of any ethnicity and separately for BRCA patients of Caucasian, African-American, or Asian ethnicity. **(D)** Boxplot showing relative expression of *TIMM17A* in normal individuals and separately for BRCA patients in stages 1, 2, 3, or 4. **(E)** Boxplot showing relative expression of *TIMM17A* in normal individuals and separately for BRCA patients with regional lymph node metastasis N0, N1, N2, or N3 tumors. **(F)** Boxplot showing relative expression of *TIMM17A* in normal individuals and separately for BRCA patients with regional lymph node metastasis N0, N1, N2 or N3 tumors. Data are mean ± SE. *P < 0.05; **P < 0.01; ***P < 0.001.

We obtained multiple *TIMM17A* expression datasets from Oncomine 4.5 databases (Rhodes et al., 2004). Both mRNA expression level and DNA copy number variation (CNV) were significantly higher in BRCA tissues than healthy breast tissues (*p* < 0.05) (Figures 1A–F). Although the fold changes were less than 2, *TIMM17A* ranked within the top 15% of differentially expressed genes based on mRNA abundance and within the top 1% based on DNA CNV (Figures 1A–F). Kaplan–Meier survival analysis using Kaplan–Meier Plotter revealed that higher *TIMM17A* expression was associated with poorer OS and shorter distant metastases-free survival (DMFS) among 1,402 BRCA cases from the Kaplan–Meier Plotter database (*p* < 0.05), and similar results were observed for GSE45255 and GSE7390 cohorts (Figures 2A–F). Among these patients, 1,402 died due to BRCA within 120 months, and patients with high expression of *TIMM17A* demonstrated worse clinical outcome. We also evaluated *TIMM17A* transcription levels in the Oncomine database, which again revealed that *TIMM17A* expression was higher in BRCA patients than healthy controls. In contrast to cancer status, *TIMM17A* expression was not associated with sex, age, ethnicity, tumor stage, tumor grade, or BRCA subtype in the UALCAN database (Figures 3A–F). Thus, *TIMM17A* expression level may serve as a prognostic indicator of BRCA.

### Genomic Alterations of *TIMM17A* in BRCA

#### Frequencies and Types of *TIMM17A* Alterations

**FIGURE 4:**
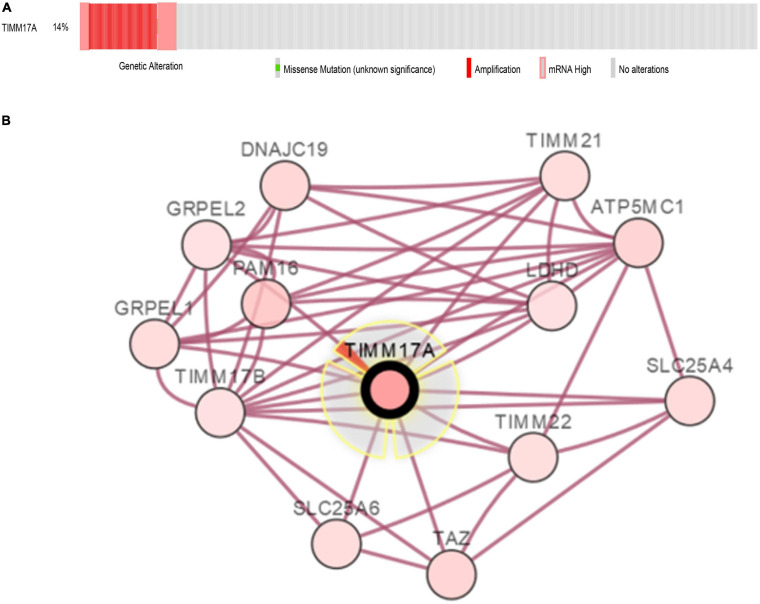
Visual summary of *TIMM17A* alterations and biological interaction networks in breast carcinoma (cBioPortal). **(A)** OncoPrint of *TIMM17A* alterations in BRCA. The OncoPrint provides an overview of genomic alterations in *TIMM17A* affecting individual samples (columns) in BRCA from the TCGA dataset. The different types of genetic alterations are indicated by unique colors. **(B)** Network view of the *TIMM17A* neighborhood in BRCA. *TIMM17A* is the seed gene (indicated with thick border), and all other genes altered in BRCA are automatically identified Darker red indicates increased alteration frequency in BRCA. The interaction types are derived from the Biological Pathway Ex-change (BioPAX). The red connection indicates proteins are members of the same complex.

The TCGA Firehose Legacy database from cBioPortal was used to assess the types and frequencies of *TIMM17A* alterations in BRCA (Gao et al., 2013). Among the 1,093 samples, *TIMM17A* was altered in 153 cases (14%) (Figure 4A). The most common alteration was amplification (111 cases, 10.16%), followed by multiple alterations (13 cases, 1.19%), and mutation only (two cases, 0.18%), while there were no samples showing mRNA upregulation (Table 1).

**TABLE 1:** Type and frequency of *TIMM17A* neighbor gene alterations in breast carcinoma (cBioPortal).

Gene symbol	Amplification	Deep deletion	Up-regulation	Down-regulation	Mutation	Multiple alternation	Total alterations
ATP5F1B	0	0	4.03	0	0.09	0.18	4.3
ATP5MC1	4.21	0.09	1.01	0	0	2.2	7.5
COQ2	0.37	0.27	3.84	0	0	0.09	4.57
DNAJC19	2.93	2.93	0	0	0.09	0.73	6.68
FXN	0.18	0.46	3.29	0	0	0.37	4.3
GRPEL1	0	0.73	4.57	0	0.09	0.27	5.67
GRPEL2	0.27	0.18	4.12	0.09	0.18	0.18	5.03
HSCB	0.64	3.75	0	0	0.09	0.27	4.76
HSPA9	0.18	0.09	3.93	0	0.27	0.18	4.67
HSPD1	0.37	2.2	0	0	0.55	0.64	3.75
LCLAT1	0.64	0.09	3.39	0	0	0.18	4.3
LDHD	0.18	1.65	2.74	0	0	0.46	5.03
PAM16	0	4.21	3.93	0	0	0.64	8.78
PMPCA	0.18	0.37	3.57	0	0.18	0.46	4.76
PMPCB	0.46	0.09	3.48	0.27	0	0.37	4.67
SLC25A4	0.27	1.56	3.66	0	0.18	0.27	5.95
SLC25A6	0.82	0.82	3.84	0	0.09	0.18	5.76
TAZ	1.28	0.27	5.12	0	0.09	0.27	7.04
TIMM10	0.27	0.18	3.2	0	0	0.37	4.03
TIMM10B	0.18	0.46	2.93	0	0	0.18	3.75
TIMM17A	10.16	2.65	0	0	0.18	1.19	14.18
TIMM17B	0.73	0.18	3.84	0	0	0.37	5.12
TIMM21	0.18	1.46	2.93	0.09	0.18	0.91	5.76
TIMM22	0.09	0.73	4.12	0	0.09	0.37	5.4
TIMM50	0.91	0.18	1.74	0	0.18	1.83	4.85
TIMM9	0.46	3.57	0	0	0.09	0.27	4.39

#### Biological Interaction Networks Associated With *TIMM17A* Alterations in BRCA

**FIGURE 5:**
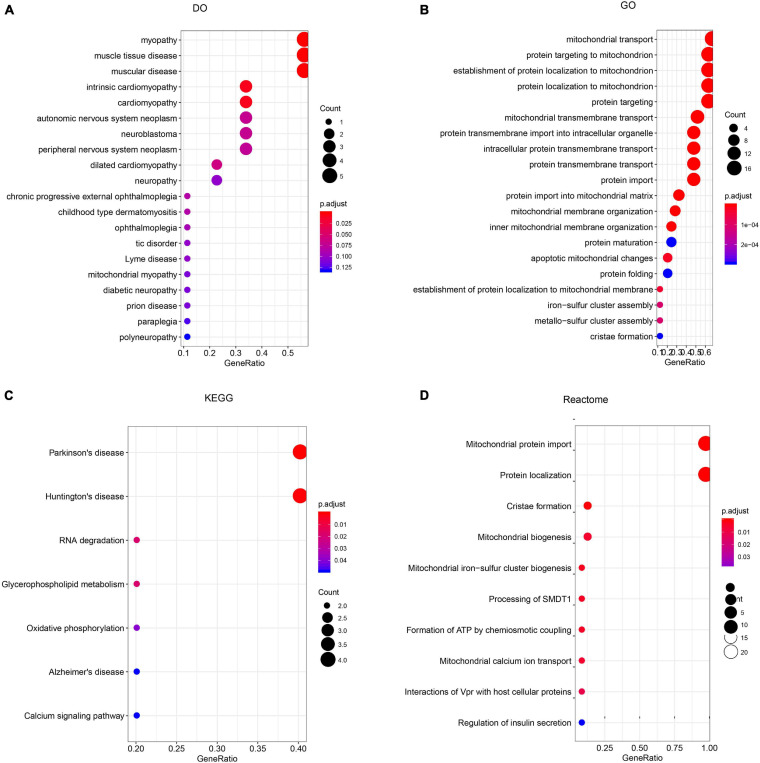
Enrichment analysis of *TIMM17A* neighbor genes altered in breast carcinoma. The bubble diagrams display the enrichment results of the top 50 *TIMM17A* neighbor genes altered in BRCA. **(A)** Disease Ontology (DO). **(B)** Gene ontology (GO). **(C)** Kyoto Encyclopedia of Genes and Genomes (KEGG). **(D)** Reactome analysis.

To construct a biological interaction network for *TIMM17A* in BRCA, we obtained neighbor genes with expression alterations at frequencies >5% using the *Network* function of cBioPortal (Figure 4B and Table 1), which retrieves networks of queried genes from public pathway databases, such as the Human Reference Protein Database, Reactome, and Memorial Sloan-Kettering Cancer Center Cancer Cell Map (Tung et al., 2016). Neighbor genes with the highest alteration frequencies were *PAM16* (8.78%), *ATP5MC1* (7.5%), and *TAZ* (7.04%). The 50 most frequently altered neighbor genes were then subjected to GO analysis, which indicated that this group was enriched in genes related to “mitochondrial transport” and “mitochondrial protein import” (Figures 5A–D).

### Enrichment Analysis of *TIMM17A* Functional Networks in BRCA

#### GO and KEGG Pathway Analysis of mRNAs Co-regulated With Mutant *TIMM17A* in BRCA

**FIGURE 6:**
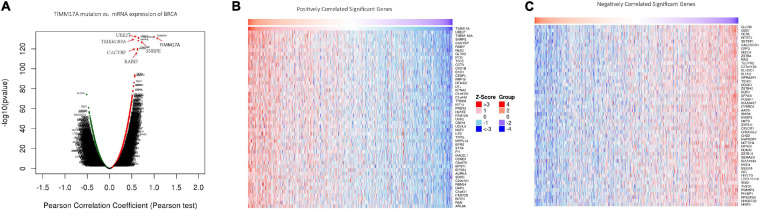
Genes differentially expressed in correlation with *TIMM17A* expression among breast carcinoma patients (LinkedOmics). **(A)** Pearson’s correlation analyses to assess the association between *TIMM17A* expression and that other genes differentially expressed in BRCA. **(B,C)** Heat maps showing genes positively and negatively correlated with *TIMM17A* expression in BRCA (TOP 50). Red indicates positively correlated genes and green indicates negatively correlated genes.

**FIGURE 7:**
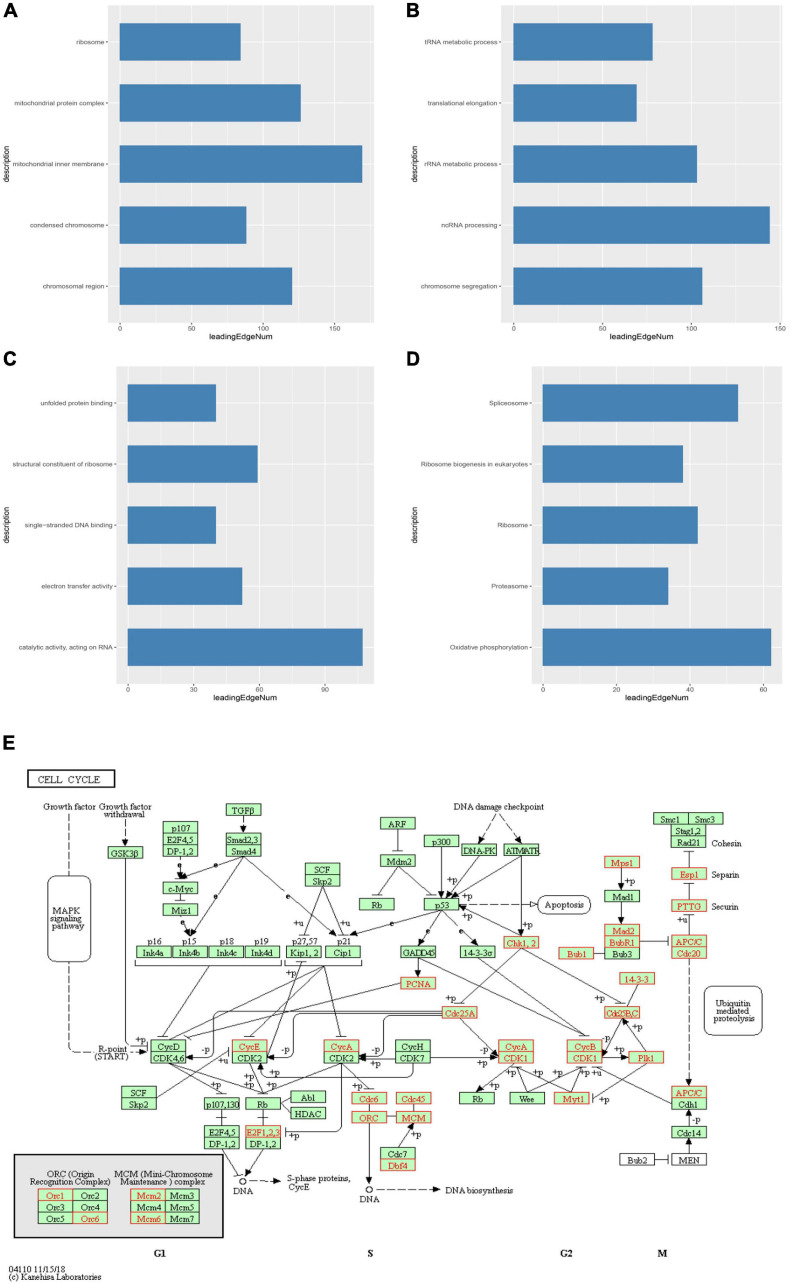
Significantly enriched GO annotations and KEGG pathways of *TIMM17A* network genes in breast carcinoma. GO annotations and KEGG pathways of genes with expression levels associated with *TIMM17A* expression in BRCA analyzed using GSEA. **(A)** Cellular components. **(B)** Biological processes. **(C)** Molecular functions. **(D)** KEGG pathway analysis. The blue column represents –log10(p). **(E)** KEGG pathway annotations of the cell cycle pathway. Red marked nodes are associated with the LeadingEdgeGene.

**FIGURE 8:**
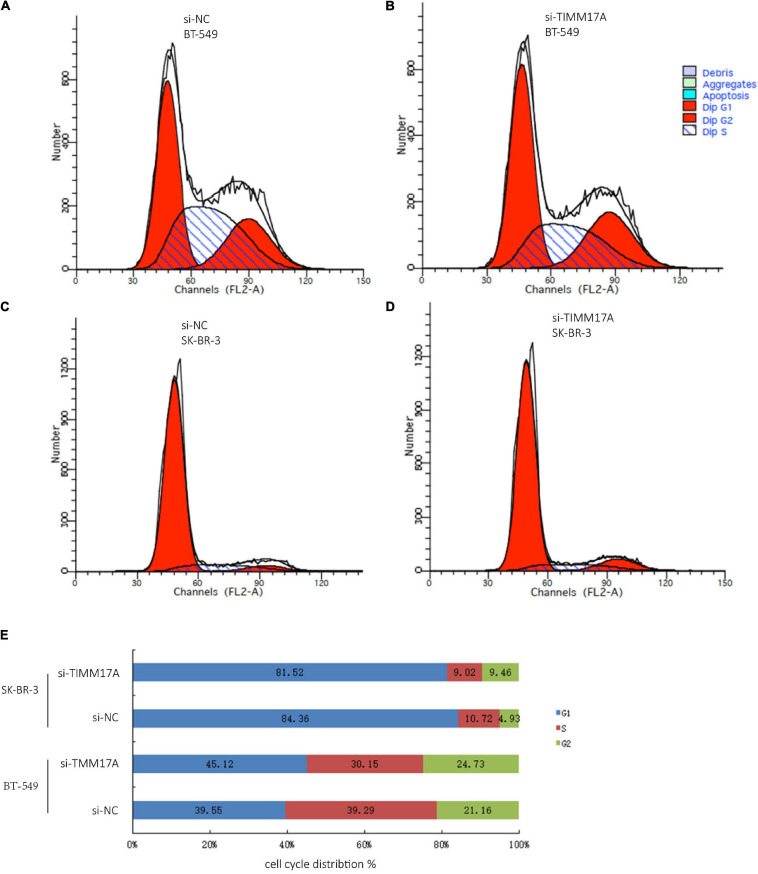
Cell cycle analysis of BRCA cells. **(A)** Cell cycle analysis of BT-549 cells by flow cytometry. **(B)** Cell cycle analysis of *TIMM17A* knockdown BT-549 cells by flow cytometry. **(C)** Cell cycle analysis of SK-BR-3 cells by flow cytometry. **(D)** Cell cycle analysis of *TIMM17A* knockdown SK-BR-3 cells by flow cytometry. **(E)** Cell cycle distribution of siRNA-SK-BR-3, NC-SK-BR-3, siRNA-BT-549 and NC-BT-549.

We then identified mRNAs demonstrating correlated expression with mutant *TIMM17A* in BRCA from TCGA using the *Function* module of LinkedOmics (Vasaikar et al., 2018). The volcano plot (Figure 6A) indicated that 7,811 genes (dark red dots) demonstrated mRNA expression levels that were positively correlated with mutant *TIMM17A* expression whereas 12,344 genes (dark green dots) demonstrated mRNA expression levels that were negatively correlated with mutant *TIMM17A* expression [false discovery rate (FDR) < 0.01]. The 50 significant gene sets exhibited both positive and negative correlations with *TIMM17A*, as shown by a heat map (Figures 6B,C). Thus, *TIMM17A* alterations in BRCA have widespread effects on the transcriptome. The statistical scatter plots for individual genes are shown in Supplementary Figures 1A–E. *TIMM17A* mutation was strongly and positively correlated with expression of *UBE2T* [positive rank #1, Pearson’s correlation (*r*) = 0.650, *p* = 5.20779257291395e−132], *TMEM183A* (*r* = 0.648, *p* = 2.22E−131), *CACYBP* (*r* = 0.628, *p* = 5.14E−121), *SNRPE* (*r* = 0.644, *p* = 6.90E−129), and *RABIF* (*r* = 0.623, *p* = 9.86E−120). GO analysis indicated that these networks are significantly enriched in genes with functions related to “mitochondrial inner membrane,” “mitochondrial protein complex,” and “chromosomal region.” Furthermore, these genes contribute to “non-coding (nc)RNA processing,” “chromosome segregation,” and “rRNA metabolic process,” and act on “RNA and structural constituents of ribosome” (Figures 7A–C and Supplementary Tables 1–3). KEGG pathway analysis indicated that these genes are related to “oxidative phosphorylation,” “spliceosome,” and “ribosome” pathways (Figures 7D,E and Supplementary Table 4). Thus, genes coregulated with mutant *TIMM17A* in BRCA participate extensively in “oxidative phosphorylation” and “cell cycle regulation.” To further validate this network-level relationship between *TIMM17A* and cell cycle regulator, we examined the association of *TIMM17A* expression with cell cycle phase distribution in BRCA cell lines by flow cytometry. The proportions of cells in S-phases were reduced by *TIMM17A* knockdown (Figures 8A–E), suggesting that *TIMM17A* serves to enhance cancer cell proliferation.

#### Kinases, miRNAs, and Transcription Factors Targeting *TIMM17A*-Associated Genes in BRCA

Finally, we analyzed the kinases, microRNAs (miRNAs), and transcription factors positively correlated with *TIMM17A* expression in BRCA by LinkedOmics. The top five most strongly associated kinases were cyclin-dependent kinase 1 (*CDK1*), polo-like kinase 1 (*PLK1*), aurora kinase B (*AURKB*), cyclin-dependent kinase 2 (*CDK2*), and checkpoint kinase 1 (*CHEK1*) (Table 2 and Supplementary Table 5). The strongly associated miRNAs included CCAGGGG (MIR-331), GAGCCTG (MIR-484), GACAATC (MIR-219), CCCAGAG (MIR-326), and AGCTCCT (MIR-28) (Table 2 and Supplementary Table 6). The associated transcription factors were mainly of the E2F transcription factor (E2F) family, including E2F_Q6, E2F1_Q6, E2F1DP2_01, E2F_02, and GGAANCGGAANY (Table 2 and Supplementary Tables 5–7). The protein–protein interaction network constructed by GeneMANIA revealed many correlations with *CDK1*, *miRNA-331*, and TF *E2F_Q6*.

**TABLE 2:** Kinases, miRNAs, and transcription factors targeting *TIMM17A*-associated gene networks in breast carcinoma (LinkedOmics).

Enriched category	Gene set	Leading Edge Num	FDR
Kinase target	Kinase_CDK1	91	0
	Kinase_PLK1	29	0
	Kinase_AURKB	31	0
	Kinase_CDK2	83	0
	Kinase_CHEK1	36	0.001906115
miRNA target	CCAGGGG,MIR-331	27	0.026158877
	GAGCCTG,MIR-484	36	0.028961613
	GACAATC,MIR-219	64	0.029428736
	CCCAGAG,MIR-326	41	0.029428736
	AGCTCCT,MIR-28	24	0.030246201
Transcription factor target	GGAANCGGAANY_UNKNOWN	40	0
	V$E2F_Q6	63	0
	V$E2F1_Q6	87	0
	SGCGSSAAA_V$E2F1DP2_01	62	0
	V$E2F_02	83	0

*LeadingEdgeNum, the number of leading edge genes; FDR, false discovery rate from Benjamini and Hochberg gene set enrichment analysis (GSEA) vs. the annotation found in Molecular Signatures Database (MSigDB) for transcription factors (TF).*

**FIGURE 9:**
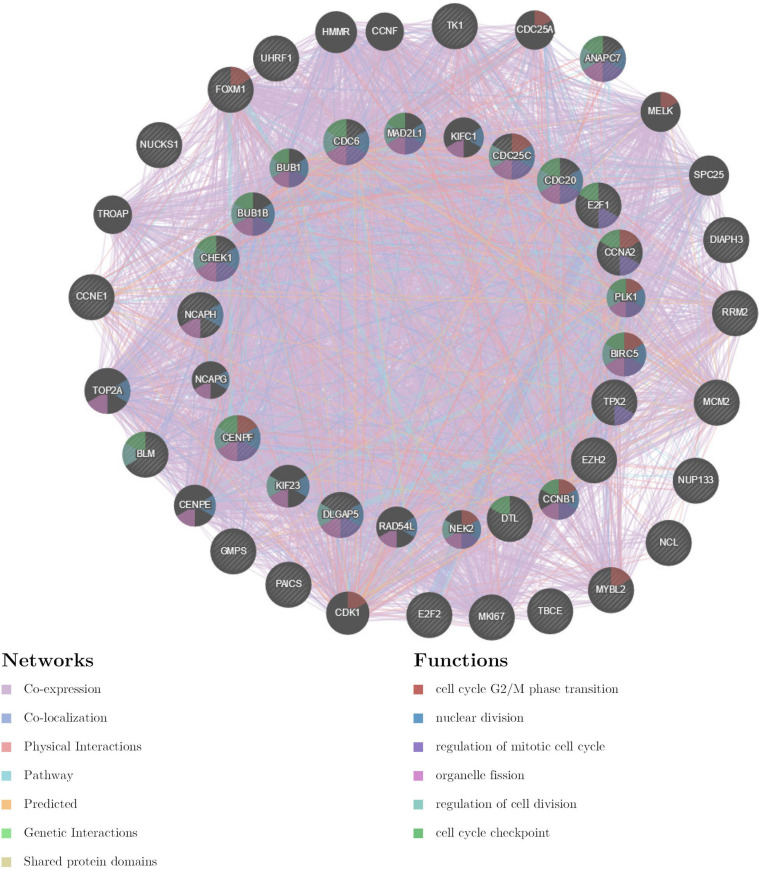
Protein–protein interaction network of ATR kinase targets (GeneMANIA). Protein–protein interaction (PPI) network and functional analysis of the gene set enriched in targets of CDK1. Different colors of the network edge indicate the bioinformatics methods applied: co-expression, co-localization, physical interactions, pathway, predicted, genetic interactions, and shared protein domains. The different colors for the network nodes indicate the biological functions of the set of enrichment genes.

**FIGURE 10:**
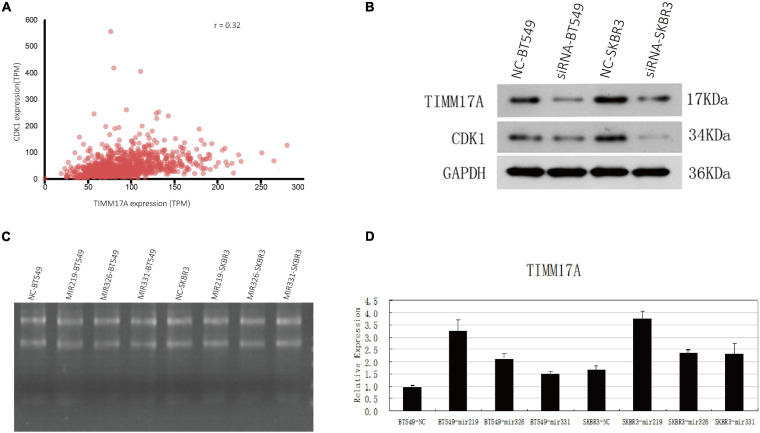
Associations of *TIMM17A* expression with *CDK1* expression and microRNAs expression levels. **(A)** Relationship between *TIMM17A* and *CDK1* expression levels. **(B)** Western blot analysis showing the effects of *TIMM17A* knock down on the expression levels of *CDK1* and *GAPDH*. **(C)** RNA electrophoretogram of miRNA 219, miRNA 326, and miRNA 331 in BRCA cells. **(D)** Expression levels of miRNA 219, miRNA 326 and miRNA 331 in *TIMM17A* knockdown BRCA cells.

The gene set enriched in *CDK1* targets is responsible mainly for regulating cell cycle G2/M-phase transition, cell division, organelle fission, and the cell cycle checkpoint (Figure 9). To validate the association between CDK1 and *TIMM17A*, we analyzed the correlation between *TIMM17A* expression and CDK1 expression both *in silico* and in BRCA cell lines. Expression of CDK1 was positively correlated with *TIMM17A* expression in theUALCAN database (Pearson’s *r* = 0.32) (Figure 10A). Furthermore, western blotting revealed that *TIMM17A* knockdown reduced CDK1expression in BRCA cell lines (Figure 10B). The gene set enriched in miRNA-331 targets is involved mainly in regulation of cell-cycle adhesion (Table 2 and Supplementary Figure 2), so we also examined the correlation between *TIMM17A* and miRNA expression in BRCA cell lines. Indeed, *TIMM17A* knockdown altered the expression level of miRNA331 as well as expression of miRNA219 and miRNA 326 in BRCA cell lines (Figures 10C,D). Similarly, the gene set enriched in transcription factor *E2F_Q6* targets is involved mainly in regulation of DNA replication, cell cycle checkpoint, and mitotic cell cycle (Supplementary Figure 3).

## Discussion

Most BRCA cases are caused by a germline mutation in a single gene or multiple genes, of which the most frequent are in *BRCA1*, *BRCA2*, and *p53* (Tung et al., 2016). Our analysis revealed an inverse association between *TIMM17A* expression level and both OS and DFMS. About 90% of all tumor recurrences and metastases occur within 10 years of first diagnosis, so we chose 10 years as the limit for survival analyses. Surprisingly, survival analyses of multiple public datasets for this period revealed that patients with high expression of *TIMM17A* have worse clinical outcome. Further subgroup analysis of multiple clinicopathological features revealed that *TIMM17A* mRNA expression was higher in BRCA patients than healthy individuals but did not differ according to sex, age, ethnicity, tumor stage, tumor grade, or BRCA subtype. The *TIMM17A* copy number was also significantly higher in BRCA tissues than normal tissues, and amplification was the most common genomic alteration with a change in copy number >2.

Expression of mutant *TIMM17A* was strongly correlated with expression levels of *UBE2T*, *TMEM183A*, *CACYBP*, *SNRPE*, and *RABIF* (top 5). The functional network of *TIMM17A* in BRCA participates extensively in chromosome segregation, oxidative phosphorylation, and cell cycle regulation. Indeed, flow cytometry demonstrated that *TIMM17A* expression was strongly associated with cell cycle phase distribution in BRCA cell lines. Specifically, *TIMM17A* knockdown reduced the proportions of cells in S-phases, suggesting that *TIMM17A* enhances the rate of proliferation. Collectively, these results demonstrate that high *TIMM17A* expression enhances BRCA aggression, potentially by promoting fasting cell cycle progression.

LinkedOmics analysis identified numerous additional network components and other gene sets with high weight coexpression and protein interactions with TIMM17A. Genes coregulated with *TIMM17A* were enriched in cell cycle modulators and targets of *CDK1*, *MIR-331*, and *E2F* family transcription factors. Activation of *CDK1* induces phosphorylation of lamin, disintegration of the nuclear fiber layer and nuclear membrane, phosphorylation of histone H1, and chromosome condensation (Haneke et al., 2020), processes required for cell division. The evolution of kinase signaling is promoted by gain or loss of phosphorylation sites in rapidly evolving regions (Holt et al., 2009). Ravindran Menon et al. (2018) found that tumor initiation is associated with regulation of transcription factor *Sox2* activity by *CDK1*, suggesting the CDK1–Sox2 pathway as a potential therapeutic target. Mitotic chromosome segregation also depends on H2B serine 6 phosphorylation by *CDK1* (Seibert et al., 2019). Unregulated CDK1-mediated phosphorylation allows the cell cycle to proceed unchecked, leading to tumor formation (Cheng et al., 2020). The current analysis identifies multiple additional CDK1-targeted pathways for potential therapeutic intervention. Moreover, we confirmed a strong correlation between *TIMM17A* and CDK1 in BRCA cells at both transcriptional and protein expression levels as evidenced by the marked CDK1 downregulation following TIMM17A knockdown.

Xuefang et al. (2020) reported that *miR-331-3p* promoted apoptosis of nasopharyngeal carcinoma cells by targeting the elf4B-PI3K-AKT pathway, and Chen et al. (2018) recently reported that *miR-331* inhibits the proliferation and invasion of melanoma cells. At the beginning of S-phase, CDK2 combines with cyclin A to inactivate E2F transcription factors which is a precondition for the completion of S-phase, while persistent E2F activity leads to apoptosis (Krek et al., 1995; Fueyo et al., 1998; Lukas et al., 1999). Therefore, selective inhibition of CDK2/cyclin A may increase *E2F* expression, which in turn could lead to S-phase arrest or apoptosis. Transcription factor E2F1 (Schuldt, 2011) plays an important role in cell cycle control and tumor suppressor gene function, and it is also the target of transforming protein of small DNA oncoviruses (Krek et al., 1995; Chen et al., 2009). These E2F family members contain multiple highly conserved domains (Osorio, 2015; Kent and Leone, 2019), including a DNA-binding domain, dimerization domain interacting with transcription factor protein (DP) regulated by differentiation, an acid amino acid-rich transactivation domain, and a tumor suppressor protein-related domain within the transactivation domain. In addition, E2F proteins E2F2 and E2F3 have cyclin-binding domains. These proteins preferentially bind to retinoblastoma protein pRb in a cell cycle-dependent manner, allowing both cell proliferation and p53-dependent or p53-independent apoptosis (Osorio, 2015). These results provide potential directions for future research on the molecular mechanisms of BRCA.

## Data Availability Statement

The original contributions presented in the study are included in the article/Supplementary Material, further inquiries can be directed to the corresponding author/s.

## Author Contributions

JJC and CXW contributed to data analysis. JJC, JYC, and LH drafted the manuscript. LH and JJC performed the experiments. ZHS and QZ designed the experiments and revised the manuscript. All of the authors critically revised the manuscript, gave final approval of the version to be published, and agreed to be accountable for all aspects of the work.

## Conflict of Interest

The authors declare that the research was conducted in the absence of any commercial or financial relationships that could be construed as a potential conflict of interest.

## Publisher’s Note

All claims expressed in this article are solely those of the authors and do not necessarily represent those of their affiliated organizations, or those of the publisher, the editors and the reviewers. Any product that may be evaluated in this article, or claim that may be made by its manufacturer, is not guaranteed or endorsed by the publisher.

## Footnotes

1
www.oncomine.org

2
http://ualcan.path.uab.edu/

3
http://cbioportal.org

4
http://www.linkedomics.org/

5
https://www.kegg.jp/

6
http://genemania.org/

## Supplementary Material

Supplementary Figure 1 Correlations between *TIMM17A* expression and UBE2T, TMEM183A, SNRPE, CACYBP, and RABIF expression (LinkedOmics). The scatter plots show Pearson’s correlations between *TIMM17A* expression and the expression levels of UBE2T **(A)**, TMEM183A **(B)**, SNRPE **(C)**, CACYBP **(D)**, and RABIF **(E)**.

Supplementary Figure 2 Protein–protein interaction network of miRNA 331 targets (GeneMANIA). Protein–protein interaction (PPI) network and functional analysis of the gene set enriched in miRNA 331 targets. Different colors of the network edge indicate the bioinformatics method applied: co-localization, co-expression, shared protein domains, pathway, and genetic interactions. The different colors for the network nodes indicate the biological functions of the set of enrichment genes.

Supplementary Figure 3 Protein–protein interaction network of transcription factor E2F_Q6 targets (GeneMANIA). Protein–protein interaction (PPI) network and functional analysis indicating the gene set enriched in transcription factor E2F_Q6 targets. Different colors of the network edge indicate the bioinformatics method applied: co-expression, physical interaction, pathway, co-localization, and predicted. The different colors for the network nodes indicate the biological functions of the set of enrichment genes.

Supplementary Table 1 Significantly enriched GO annotations (cellular components) of *TIMM17A* in breast carcinoma (LinkedOmics).

Supplementary Table 2 Significantly enriched GO annotations (biological processes) of *TIMM17A* in breast carcinoma (LinkedOmics).

Supplementary Table 3 Significantly enriched GO annotations (molecular functions) of *TIMM17A* in breast carcinoma (LinkedOmics).

Supplementary Table 4 Significantly enriched KEGG pathway annotations of *TIMM17A* in breast carcinoma (LinkedOmics).

Supplementary Table 5 Significantly enriched kinase-target networks of *TIMM17A* in breast carcinoma (LinkedOmics).

Supplementary Table 6 Significantly enriched miRNA-target networks of *TIMM17A* in breast carcinoma (LinkedOmics).

Supplementary Table 7 Significantly enriched transcription factor-target networks of *TIMM17A* in breast carcinoma (LinkedOmics).

Supplementary Table 8 The sequences of primers used for qPCR.

## References

[B1] Ahn S. Kim H. J. Kang E. Kim E. K. Kim S. H. Kim J. H. (2020). Genomic profiling of multiple breast cancer reveals inter-lesional heterogeneity. *Br. J. Cancer* 122 697–704. 10.1038/s41416-019-0713-1 31929516 PMC7054255

[B2] Bomer U. Rassow J. Zufall N. Pfanner N. Meijer M. Maarse A. C. (1996). The preprotein translocase of the inner mitochondrial membrane: evolutionary conservation of targeting and assembly of Tim17. *J. Mol. Biol.* 262 389–395. 10.1006/jmbi.1996.0522 8893850

[B3] Bray F. Ferlay J. Soerjomataram I. Siegel R. L. Torre L. A. Jemal A. (2018). Global cancer statistics 2018: GLOBOCAN estimates of incidence and mortality worldwide for 36 cancers in 185 countries. *CA Cancer J. Clin.* 68 394–424. 10.3322/caac.21492 30207593

[B4] Chandrashekar D. S. Bashel B. Balasubramanya S. A. H. Creighton C. J. Ponce-Rodriguez I. Chakravarthi B. V. S. K. (2017). UALCAN: a portal for facilitating tumor subgroup gene expression and survival analyses. *Neoplasia* 19 649–658. 10.1016/j.neo.2017.05.002 28732212 PMC5516091

[B5] Chen H. Z. Tsai S. Y. Leone G. (2009). Emerging roles of E2Fs in cancer: an exit from cell cycle control. *Nat. Rev. Cancer* 9 785–797. 10.1038/nrc2696 19851314 PMC3616489

[B6] Chen L. Ma G. Cao X. An X. Liu X. (2018). MicroRNA-331 Inhibits Proliferation and Invasion of Melanoma Cells by Targeting Astrocyte-Elevated Gene-1. *Oncol. Res.* 26 1429–1437. 10.3727/096504018x15186047251584 29510779 PMC7844642

[B7] Cheng C. Yun F. Ullah S. Yuan Q. (2020). Discovery of novel cyclin-dependent kinase (CDK) and histone deacetylase (HDAC) dual inhibitors with potent in vitro and in vivo anticancer activity. *Eur. J. Med. Chem.* 189:112073. 10.1016/j.ejmech.2020.112073 31991336

[B8] Fueyo J. Gomez-Manzano C. Yung W. K. Liu T. J. Alemany R. McDonnell T. J. (1998). Overexpression of E2F-1 in glioma triggers apoptosis and suppresses tumor growth in vitro and in vivo. *Nat. Med.* 4 685–690. 10.1038/nm0698-685 9623977

[B9] Gao J. Aksoy B. A. Dogrusoz U. Dresdner G. Gross B. Sumer S. O. (2013). Integrative analysis of complex cancer genomics and clinical profiles using the cBioPortal. *Sci. Signal.* 6:pl1. 10.1126/scisignal.2004088 23550210 PMC4160307

[B10] Garrido-Castro A. C. Winer E. P. (2018). Predicting breast cancer therapeutic response. *Nat. Med.* 24 535–537. 10.1038/s41591-018-0033-7 29736021

[B11] Haneke K. Schott J. Lindner D. Hollensen A. K. Damgaard C. K. Mongis C. (2020). CDK1 couples proliferation with protein synthesis. *J. Cell Biol.* 219:e201906147. 10.1083/jcb.201906147 32040547 PMC7054999

[B12] Harbeck N. Gnant M. (2017). Breast cancer. *Lancet* 389 1134–1150. 10.1016/s0140-6736(16)31891-8 27865536

[B13] Hayes M. D. W. D. N. (2010). ConsensusClusterPlus: a class discovery tool with confidence assessments and item tracking. *Bioinformatics* 26 1572–1573. 10.1093/bioinformatics/btq170 20427518 PMC2881355

[B14] Holt L. J. Tuch B. B. Villén J. Johnson A. D. Gygi S. P. Morgan D. O. (2009). Global analysis of Cdk1 substrate phosphorylation sites provides insights into evolution. *Science* 325 1682–1686. 10.1126/science.1172867 19779198 PMC2813701

[B15] Kanehisa M. Furumichi M. Tanabe M. Sato Y. Morishima K. (2017). KEGG: new perspectives on genomes, pathways, diseases and drugs. *Nucleic Acids Res.* 45 D353–D361. 10.1093/nar/gkw1092 27899662 PMC5210567

[B16] Kannangai R. Vivekanandan P. Martinez-Murillo F. Choti M. Torbenson M. (2007). Fibrolamellar carcinomas show overexpression of genes in the RAS, MAPK, PIK3, and xenobiotic degradation pathways. *Hum. Pathol.* 38 639–644. 10.1016/j.humpath.2006.07.019 17367606

[B17] Kent L. N. Leone G. (2019). The broken cycle: E2F dysfunction in cancer. *Nat. Rev. Cancer* 19 326–338. 10.1038/s41568-019-0143-7 31053804

[B18] Krek W. Xu G. Livingston D. M. (1995). Cyclin A-kinase regulation of E2F-1 DNA binding function underlies suppression of an S phase checkpoint. *Cell* 83 1149–1158. 10.1016/0092-8674(95)90141-8 8548802

[B19] Krug K. Jaehnig E. J. Satpathy S. Blumenberg L. Karpova A. Anurag M. (2020). Proteogenomic Landscape of Breast Cancer Tumorigenesis and Targeted Therapy. *Cell* 183 1436–1456.e31. 10.1016/j.cell.2020.10.036 33212010 PMC8077737

[B20] Liang Y. Zhang H. Song X. Yang Q. (2020). Metastatic heterogeneity of breast cancer: molecular mechanism and potential therapeutic targets. *Semin. Cancer Biol.* 60 14–27. 10.1016/j.semcancer.2019.08.012 31421262

[B21] Lukas C. Sørensen C. S. Kramer E. Santoni-Rugiu E. Lindeneg C. Peters J. M. (1999). Accumulation of cyclin B1 requires E2F and cyclin-A-dependent rearrangement of the anaphase-promoting complex. *Nature* 401 815–818. 10.1038/44611 10548110

[B22] Mishra N. K. Southekal S. Guda C. (2019). Survival Analysis of Multi-Omics Data Identifies Potential Prognostic Markers of Pancreatic Ductal Adenocarcinoma. *Front. Genet.* 10:624. 10.3389/fgene.2019.00624 31379917 PMC6659773

[B23] Okano M. Oshi M. Butash A. L. Katsuta E. Tachibana K. Saito K. (2019). Triple-Negative breast cancer with high levels of annexin a1 expression is associated with mast cell infiltration, inflammation, and angiogenesis. *Int. J. Mol. Sci.* 20:4197. 10.3390/ijms20174197 31461932 PMC6747082

[B24] Osorio J. (2015). Cell cycle: repurposing MYC and E2F in the absence of RB. *Nat. Rev. Mol. Cell Biol.* 16 516–517. 10.1038/nrm4044 26265406

[B25] Radvanyi L. G. (2018). Targeting the cancer mutanome of breast cancer. *Nat. Med.* 24 703–704. 10.1038/s41591-018-0065-z 29867234

[B26] Ravindran Menon D. Luo Y. Arcaroli J. J. Liu S. KrishnanKutty L. N. Osborne D. G. (2018). CDK1 Interacts with Sox2 and Promotes Tumor Initiation in Human Melanoma. *Cancer Res.* 78 6561–6574. 10.1158/0008-5472.can-18-0330 30297536 PMC6279496

[B27] Rhodes D. R. Yu J. Shanker K. Deshpande N. Varambally R. Ghosh D. (2004). ONCOMINE: a cancer microarray database and integrated data-mining platform. *Neoplasia* 6 1–6. 10.1016/s1476-5586(04)80047-2 15068665 PMC1635162

[B28] Salhab M. Patani N. Jiang W. Mokbel K. (2012). High TIMM17A expression is associated with adverse pathological and clinical outcomes in human breast cancer. *Breast Cancer* 19 153–160. 10.1007/s12282-010-0228-3 20972741

[B29] Schuldt A. (2011). Cell cycle: E2F1 ensures the endocycle. *Nat. Rev. Mol. Cell Biol.* 12:768. 10.1038/nrm3232 22086370

[B30] Seibert M. Krüger M. Watson N. A. Sen O. Daum J. R. Slotman J. A. (2019). CDK1-mediated phosphorylation at H2B serine 6 is required for mitotic chromosome segregation. *J. Cell Biol.* 218 1164–1181. 10.1083/jcb.201806057 30765437 PMC6446833

[B31] Tung N. Lin N. U. Kidd J. Allen B. A. Singh N. Wenstrup R. J. (2016). Frequency of Germline Mutations in 25 Cancer Susceptibility Genes in a Sequential Series of Patients With Breast Cancer. *J. Clin. Oncol.* 34 1460–1468. 10.1200/jco.2015.65.0747 26976419 PMC4872307

[B32] Vasaikar S. V. Straub P. Wang J. Zhang B. (2018). LinkedOmics: analyzing multi-omics data within and across 32 cancer types. *Nucleic Acids Res.* 46 D956–D963. 10.1093/nar/gkx1090 29136207 PMC5753188

[B33] Warde-Farley D. Donaldson S. L. Comes O. Zuberi K. Badrawi R. Chao P. (2010). The GeneMANIA prediction server: biological network integration for gene prioritization and predicting gene function. *Nucleic Acids Res.* 38 W214–W220. 10.1093/nar/gkq537 20576703 PMC2896186

[B34] Xu X. Qiao M. Zhang Y. Jiang Y. Wei P. Yao J. (2010). Quantitative proteomics study of breast cancer cell lines isolated from a single patient: discovery of TIMM17A as a marker for breast cancer. *Proteomics* 10 1374–1390. 10.1002/pmic.200900380 20198662

[B35] Xuefang Z. Ruinian Z. Liji J. Chun Z. Qiaolan Z. Jun J. (2020). miR-331-3p Inhibits Proliferation and Promotes Apoptosis of Nasopharyngeal Carcinoma Cells by Targeting elf4B-PI3K-AKT Pathway. *Technol. Cancer Res. Treat.* 19:1533033819892251. 10.1177/1533033819892251 31984860 PMC6985969

[B36] Yang X. Si Y. Tao T. Martin T. A. Cheng S. Yu H. (2016). The Impact of TIMM17A on Aggressiveness of Human Breast Cancer Cells. *Anticancer Res.* 36 1237–1241. 26977020

[B37] Yeo S. K. Guan J. L. (2017). Breast Cancer: multiple subtypes within a Tumor? *Trends Cancer* 3 753–760. 10.1016/j.trecan.2017.09.001 29120751 PMC5802368

[B38] Zacharakis N. Chinnasamy H. Black M. Xu H. Lu Y. C. Zheng Z. (2018). Immune recognition of somatic mutations leading to complete durable regression in metastatic breast cancer. *Nat. Med.* 24 724–730. 10.1038/s41591-018-0040-8 29867227 PMC6348479

[B39] Zardavas D. Irrthum A. Swanton C. Piccart M. (2015). Clinical management of breast cancer heterogeneity. *Nat. Rev. Clin. Oncol.* 12 381–394. 10.1038/nrclinonc.2015.73.8 25895611

