# Supplementary figures and images for: A TIMM17A Regulatory Network Contributing to Breast Cancer
Source: Front Genet. 2021 Aug 5;12:658154. doi: 10.3389/fgene.2021.658154 (PMC8375323; doi:10.3389/fgene.2021.658154)

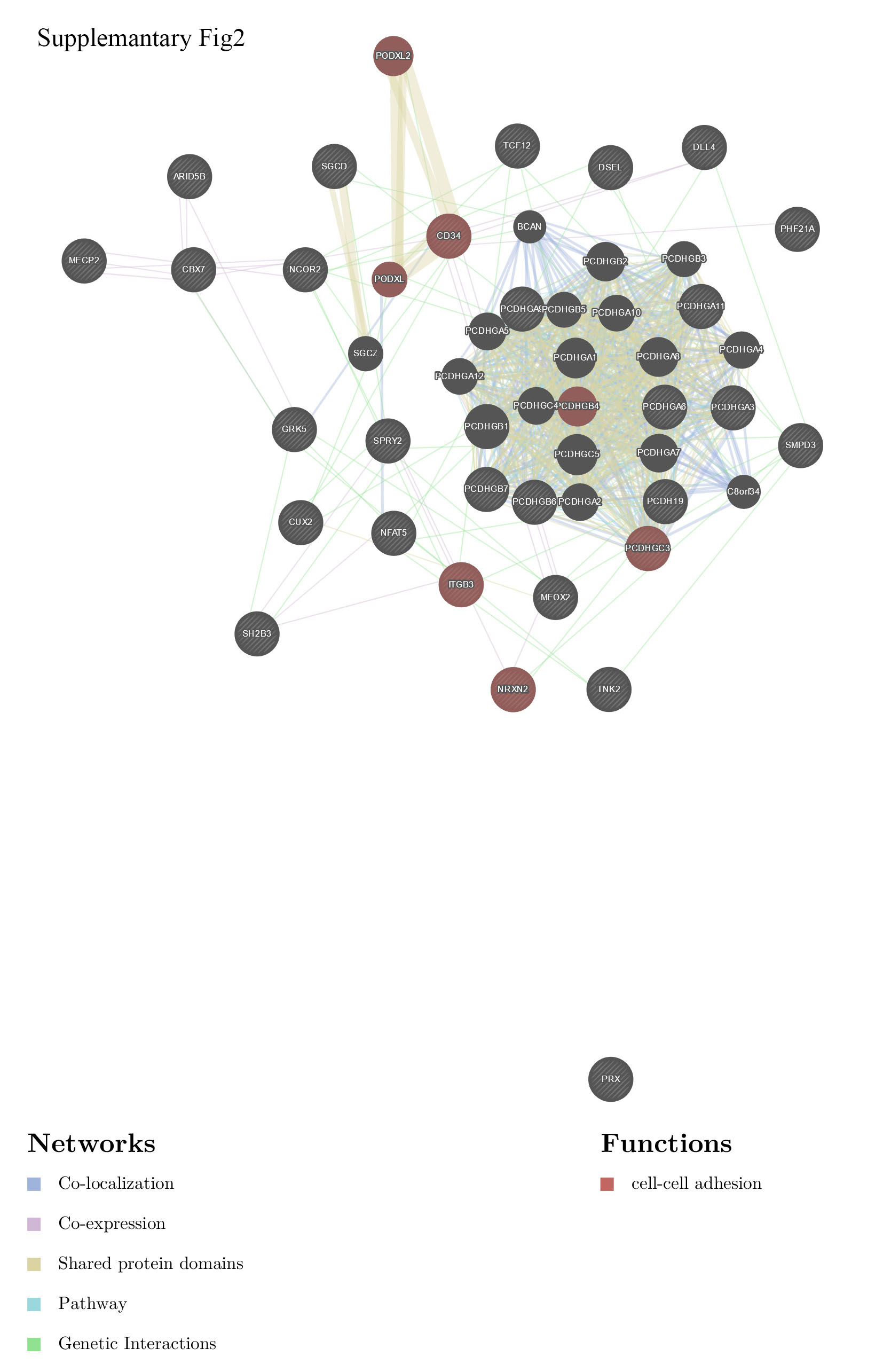

Supplement: Supplementary Figure 1 — Correlations between TIMM17A expression and UBE2T, TMEM183A, SNRPE, CACYBP, and RABIF expression (LinkedOmics). The scatter plots show Pearson’s correlations between TIMM17A expression and the expression levels of UBE2T (A), TMEM183A (B), SNRPE (C), CACYBP (D), and RABIF (E). [file Image_1.JPEG]

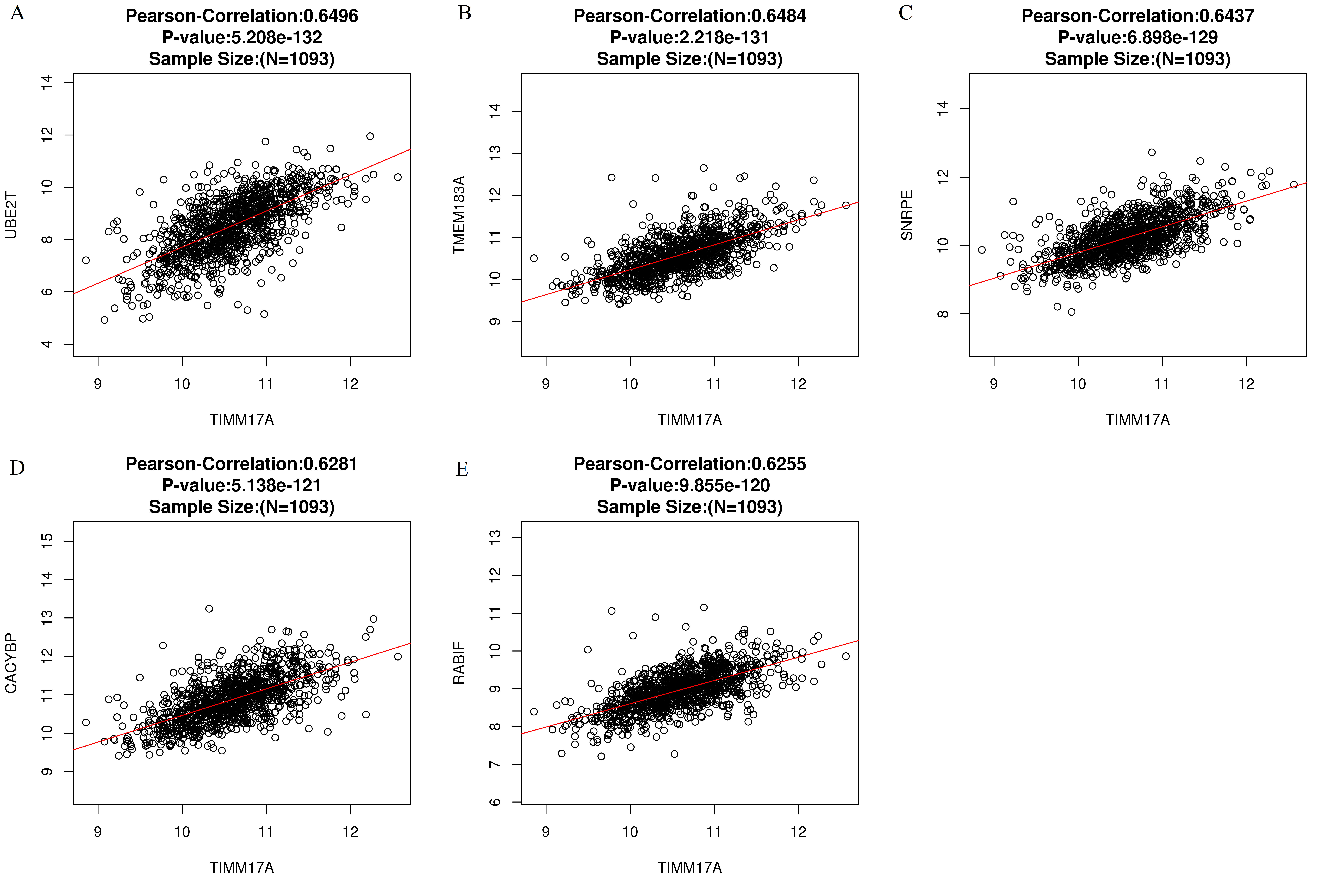

Supplement: Supplementary Figure 2 — Protein–protein interaction network of miRNA 331 targets (GeneMANIA). Protein–protein interaction (PPI) network and functional analysis of the gene set enriched in miRNA 331 targets. Different colors of the network edge indicate the bioinformatics method applied: co-localization, co-expression, shared protein domains, pathway, and genetic interactions. The different colors for the network nodes indicate the biological functions of the set of enrichment genes. [file Image_2.PNG]

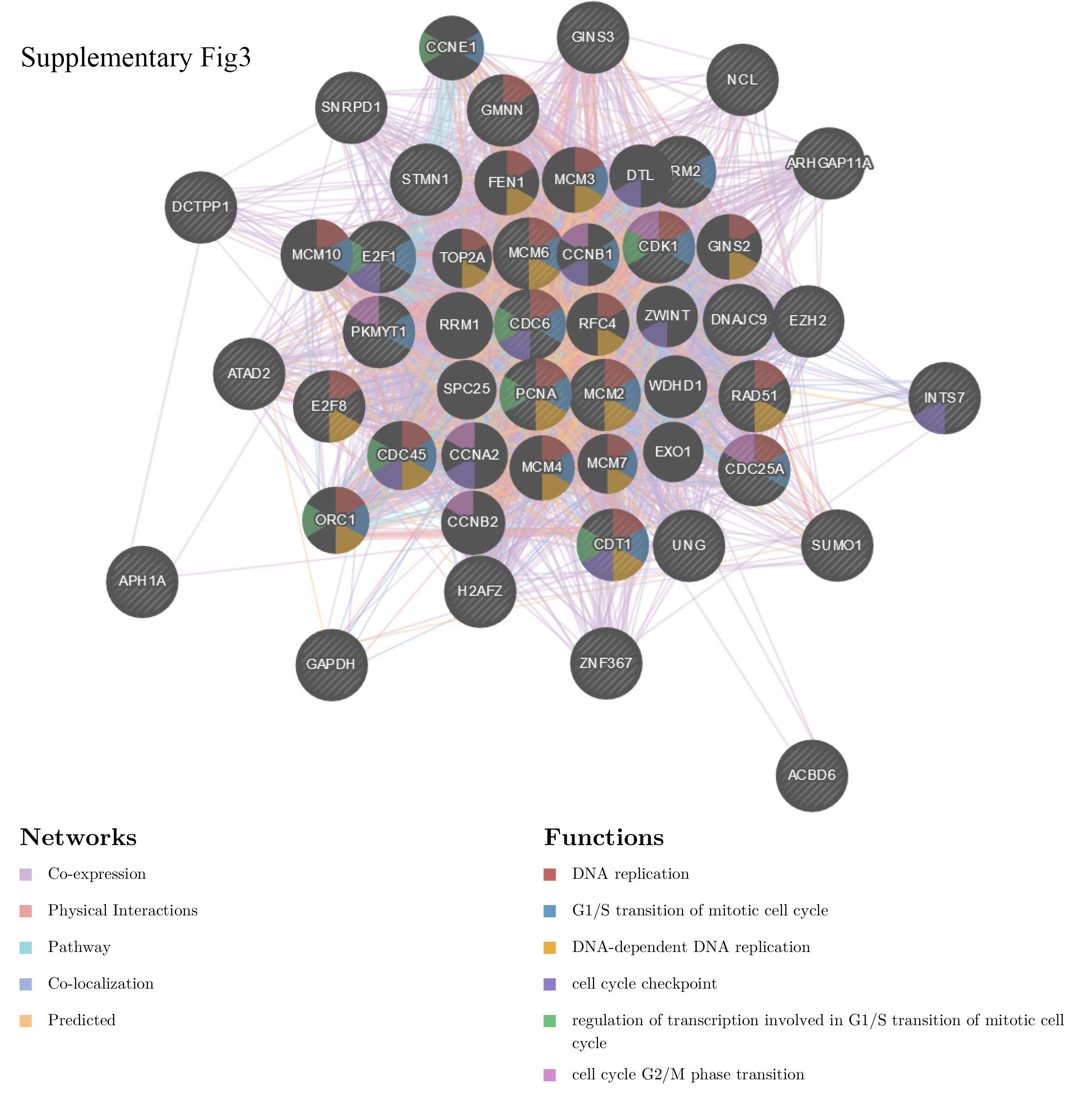

Supplement: Supplementary Figure 3 — Protein–protein interaction network of transcription factor E2F_Q6 targets (GeneMANIA). Protein–protein interaction (PPI) network and functional analysis indicating the gene set enriched in transcription factor E2F_Q6 targets. Different colors of the network edge indicate the bioinformatics method applied: co-expression, physical interaction, pathway, co-localization, and predicted. The different colors for the network nodes indicate the biological functions of the set of enrichment genes. [file Image_3.JPEG]
