# Supplementary material for: A TIMM17A Regulatory Network Contributing to Breast Cancer
Source: Front Genet. 2021 Aug 5;12:658154. doi: 10.3389/fgene.2021.658154 (PMC8375323; doi:10.3389/fgene.2021.658154)
Supplement: Supplementary Table 1 — Significantly enriched GO annotations (cellular components) of TIMM17A in breast carcinoma (LinkedOmics). [file Table_1.DOCX]

**Supplementary Table 1. Significantly enriched GO annotations (cellular components) of TIMM17A in breast carcinoma (LinkedOmics)**

| description | leadingEdgeNum | FDR | LeadingEdgeGene |  |
| --- | --- | --- | --- | --- |
| mitochondrial protein complex | 126 | 0 | AFG3L2;APOO;BCS1L;CHCHD1;CHCHD3;COX5A;COX5B;COX6A1;COX7A2;COX7A2L;COX7C;CYC1;DAP3;DLAT;DNA2;GRPEL1;GRPEL2;HSD17B10;IMMP1L;IMMT;MCCC1;MFN1;MRPL11;MRPL12;MRPL13;MRPL14;MRPL15;MRPL17;MRPL18;MRPL19;MRPL2;MRPL21;MRPL22;MRPL24;MRPL27;MRPL3;MRPL30;MRPL32;MRPL33;MRPL35;MRPL36;MRPL37;MRPL38;MRPL39;MRPL4;MRPL42;MRPL44;MRPL46;MRPL47;MRPL48;MRPL50;MRPL51;MRPL52;MRPL55;MRPL9;MRPS10;MRPS11;MRPS12;MRPS14;MRPS15;MRPS16;MRPS17;MRPS18A;MRPS18B;MRPS18C;MRPS2;MRPS21;MRPS22;MRPS24;MRPS28;MRPS33;MRPS35;MRPS5;MRPS6;MRPS7;MRPS9;MTX1;MTX2;NDUFA1;NDUFA12;NDUFA4;NDUFA6;NDUFA7;NDUFA8;NDUFA9;NDUFAB1;NDUFB10;NDUFB11;NDUFB2;NDUFB3;NDUFB4;NDUFB5;NDUFB6;NDUFB9;NDUFS1;NDUFS2;NDUFS3;NDUFS5;NDUFS6;NDUFV3;PNPT1;PPIF;ROMO1;SDHA;SDHB;SDHC;SUCLG1;SUPV3L1;TIMM10;TIMM17A;TIMM17B;TIMM50;TIMM8B;TIMM9;TOMM20;TOMM22;TOMM40;TOMM40L;TOMM5;TOMM6;UQCR10;UQCRB;UQCRFS1;UQCRH;UQCRHL;VDAC1 |  |
| chromosomal region | 120 | 0 | AHCTF1;AURKA;AURKB;BIRC5;BLM;BUB1;BUB1B;CBX1;CBX3;CCNB1;CDC73;CDCA5;CDCA8;CDK1;CDK2;CDT1;CEBPB;CENPA;CENPE;CENPF;CENPH;CENPI;CENPK;CENPL;CENPM;CENPN;CENPO;CENPQ;CENPW;CFDP1;CHEK1;CHEK2;CKAP5;DCTN5;DNA2;DNMT3A;DSCC1;DSN1;DYNLL1;ERCC6L;ESCO2;FBXO28;FEN1;GAR1;H2AFX;H2AFY;H3F3A;HELLS;HJURP;HNRNPA2B1;HNRNPU;HSF1;KIF18A;KIF22;KIF2C;KNTC1;MAD2L1;MCM2;MCM3;MCM4;MCM6;MCM7;MSH2;MTBP;NAT10;NBN;NCAPD2;NCAPG;NDC80;NEK2;NGDN;NHP2;NSL1;NSMCE2;NUF2;NUP107;NUP133;NUP37;NUP85;OIP5;PARP1;PCNA;PIF1;PLK1;POLR2B;PPP1CC;PPP2R5A;PRKDC;PSEN2;PTGES3;RAD21;RAD51;RECQL4;RNF8;SEC13;SEH1L;SKA1;SKA2;SKA3;SMC1B;SMC6;SPAG5;SPC24;SPC25;SSB;SUGT1;SUMO3;SUV39H1;SUV39H2;TERF1;THOC3;THOC7;TPR;TTK;XPO1;XRCC6;ZNF207;ZW10;ZWILCH;ZWINT | |
| condensed chromosome | 88 | 0 | AHCTF1;AURKA;AURKB;BANF1;BIRC5;BLM;BRCA1;BUB1;BUB1B;CBX3;CCNB1;CDCA5;CDK2;CDT1;CEBPB;CENPA;CENPE;CENPF;CENPH;CENPK;CENPM;CENPN;CENPO;CENPW;CFDP1;CHEK1;CHMP1A;CKAP5;DCTN5;DSN1;ERCC6L;FANCD2;FBXO28;H2AFX;H2AFY;HJURP;HMGB2;HNRNPU;HSF1;HUS1;KIF2C;KIFAP3;KNTC1;LRPPRC;MAD2L1;MKI67;NCAPD2;NCAPG;NCAPG2;NCAPH;NDC80;NEK2;NSL1;NSMCE2;NUF2;NUP107;NUP133;NUP37;NUP85;PES1;PLK1;PPP1CC;RAD1;RAD21;RAD51;RCC1;RRS1;SEH1L;SKA1;SKA2;SKA3;SMC1B;SMC2;SMC4;SMC6;SPAG5;SPC24;SPC25;SUV39H1;TOP2A;TOPBP1;TUBG1;UBE2I;XRCC4;ZNF207;ZW10;ZWILCH;ZWINT | |
| mitochondrial inner membrane | 169 | 0 | ABCB10;ACAD9;AFG3L2;AIFM1;APOO;BCS1L;CHCHD1;CHCHD3;COQ3;COQ5;COQ9;COX10;COX16;COX5A;COX5B;COX6A1;COX6B1;COX7A2;COX7A2L;COX7B;COX7C;CYC1;CYCS;DAP3;DHODH;ERAL1;GHITM;GOT2;GRPEL1;GRPEL2;HCCS;HSPD1;IFI6;IMMP1L;IMMT;L2HGDH;MRPL1;MRPL11;MRPL12;MRPL13;MRPL14;MRPL15;MRPL17;MRPL18;MRPL19;MRPL2;MRPL21;MRPL22;MRPL24;MRPL27;MRPL3;MRPL30;MRPL32;MRPL33;MRPL35;MRPL36;MRPL37;MRPL38;MRPL39;MRPL4;MRPL42;MRPL44;MRPL46;MRPL47;MRPL48;MRPL50;MRPL51;MRPL52;MRPL55;MRPL9;MRPS10;MRPS11;MRPS12;MRPS14;MRPS15;MRPS16;MRPS17;MRPS18A;MRPS18B;MRPS18C;MRPS2;MRPS21;MRPS22;MRPS23;MRPS24;MRPS25;MRPS28;MRPS33;MRPS35;MRPS5;MRPS6;MRPS7;MRPS9;MRS2;MTCH1;MTCH2;NDUFA1;NDUFA12;NDUFA4;NDUFA6;NDUFA7;NDUFA8;NDUFA9;NDUFAB1;NDUFAF2;NDUFAF4;NDUFB10;NDUFB11;NDUFB2;NDUFB3;NDUFB4;NDUFB5;NDUFB6;NDUFB9;NDUFS1;NDUFS2;NDUFS3;NDUFS5;NDUFS6;NDUFV2;NDUFV3;OPA1;PARL;PHB;PPIF;ROMO1;RSAD2;SCO1;SCO2;SDHA;SDHB;SDHC;SFXN4;SHMT2;SIRT5;SLC25A1;SLC25A10;SLC25A13;SLC25A19;SLC25A3;SLC25A32;SLC25A33;SLC25A39;SLC25A43;SLC25A44;SLC25A5;STOML2;TIMM10;TIMM17A;TIMM17B;TIMM50;TIMM8A;TIMM8B;TIMM9;TMEM14C;TMEM177;TMEM65;TMEM70;TOMM40;TRAP1;TYMS;UQCR10;UQCR11;UQCRB;UQCRFS1;UQCRH;UQCRHL;UQCRQ;YME1L1 | |
| ribosome | 84 | 0 | ABCF1;CANX;CHCHD1;DAP3;DENR;DHX9;EIF2AK2;EIF3H;EIF4G1;FMR1;HSPA14;LARP4B;MCTS1;MRPL1;MRPL11;MRPL12;MRPL13;MRPL14;MRPL15;MRPL17;MRPL18;MRPL19;MRPL2;MRPL21;MRPL22;MRPL24;MRPL27;MRPL3;MRPL32;MRPL33;MRPL35;MRPL36;MRPL37;MRPL38;MRPL39;MRPL4;MRPL42;MRPL44;MRPL46;MRPL47;MRPL48;MRPL50;MRPL51;MRPL52;MRPL55;MRPL9;MRPS10;MRPS11;MRPS12;MRPS14;MRPS15;MRPS16;MRPS17;MRPS18A;MRPS18B;MRPS18C;MRPS2;MRPS21;MRPS22;MRPS23;MRPS24;MRPS25;MRPS28;MRPS33;MRPS35;MRPS5;MRPS6;MRPS7;MRPS9;NAA10;NDUFA7;NDUFAB1;NUFIP2;PNPT1;RPL22L1;RPL26L1;RPL30;RPL38;RPL39;RPL39L;RPL7;RPL7L1;SRP68;ZC3H15 | |

Abbreviations: LeadingEdgeNum, the number of leadingedge genes; FDR, false discovery rate from Benjamini and Hochberg from gene set enrichment analysis (GSEA).
