# Supplementary material for: A TIMM17A Regulatory Network Contributing to Breast Cancer
Source: Front Genet. 2021 Aug 5;12:658154. doi: 10.3389/fgene.2021.658154 (PMC8375323; doi:10.3389/fgene.2021.658154)
Supplement: Supplementary Table 2 — Significantly enriched GO annotations (biological processes) of TIMM17A in breast carcinoma (LinkedOmics). [file Table_2.DOCX]

**Supplementary Table 2. Significantly enriched GO annotations (biological processes) of TIMM17A in breast carcinoma (LinkedOmics).**

| Description | LeadingEdgeGene | |
| --- | --- | --- |
| chromosome segregation | | ACTR3;ANAPC11;ARL8A;AURKB;BIRC5;BLM;BRCA1;BRIP1;BUB1;BUB1B;CCNB1;CCNE1;CCNE2;CDC20;CDC6;CDCA5;CDCA8;CDT1;CENPE;CENPF;CENPN;CENPQ;CENPW;CEP55;CHMP1A;CHMP4C;CHMP5;DLGAP5;DSCC1;DSN1;ECT2;EME1;ESCO2;ESPL1;FAM83D;FANCD2;FBXO5;FEN1;GEN1;H2AFY;HJURP;HNRNPU;KIF14;KIF18A;KIF18B;KIF22;KIF23;KIF2C;KIF4A;KIF4B;KIFC1;KPNB1;MAD2L1;MKI67;MSTO1;NAA10;NAA50;NCAPD2;NCAPG;NCAPH;NDC80;NEK2;NSL1;NSMCE2;NUF2;NUP37;NUSAP1;OIP5;PLK1;PRC1;PSMG2;PSRC1;PTTG1;RACGAP1;RAD18;RAD21;RAD51C;RAN;RCC1;RMI1;RRS1;SEH1L;SFPQ;SKA1;SKA2;SKA3;SLC25A5;SMC1B;SMC2;SMC4;SMC6;SPAG5;SPC25;SRPK1;TACC3;TERF1;TLK2;TOP2A;TPR;TRIP13;TTK;TUBG1;UBE2I;ZNF207;ZW10;ZWINT |
| ncRNA processing | | AARS;ABT1;ADAR;BMS1;BOP1;BYSL;C1D;CDK5RAP1;CLP1;CPSF3;DCAF13;DDX1;DDX27;DDX47;DDX49;DDX52;DDX56;DKC1;DPH3;DUS1L;EBNA1BP2;EIF4A3;EMG1;ERI3;ESF1;EXOSC2;EXOSC3;EXOSC4;EXOSC5;EXOSC6;EXOSC7;EXOSC8;EXOSC9;FBL;FTSJ1;FTSJ3;GAR1;GRSF1;GTF2H5;GTPBP4;HEATR1;HNRNPA2B1;HSD17B10;IMP4;INTS2;INTS7;INTS8;ISG20L2;KARS;LAGE3;LAS1L;LSM6;LYAR;METTL1;METTL2A;METTL2B;METTL6;MOCS3;MPHOSPH10;MPHOSPH6;MRPL1;MRPL44;MRPS11;MRPS9;MRTO4;NAT10;NCBP1;NGDN;NHP2;NOC4L;NOL10;NOL11;NOLC1;NOP10;NOP14;NOP2;NOP56;NOP58;NPM3;NSUN2;NSUN5;NVL;PA2G4;PDCD11;PES1;POLR3K;POP1;POP4;POP7;PRKRA;PUS1;PUS7;RCL1;RIOK1;RPF2;RPL7;RPL7L1;RPP21;RPP25;RPP30;RPP38;RPP40;RPS21;RPS27;RRP1;RRP15;RRP1B;RRP9;RRS1;RSL1D1;SEPHS1;SEPHS2;SSB;SUV39H1;TARBP1;TEX10;TFB2M;THUMPD2;THUMPD3;TP53RK;TPRKB;TRIT1;TRMT12;TRMT6;TRNT1;TSEN15;TSEN54;TSR1;USP14;UTP14A;UTP15;UTP18;UTP20;UTP23;UTP6;WDR12;WDR3;WDR4;WDR43;WDR46;WDR74;WDR75;ZBTB8OS;ZNHIT3 |
| tRNA metabolic process | | AARS;AIMP1;AIMP2;CARS;CDK5RAP1;CLP1;DARS2;DDX1;DPH3;DUS1L;EEF1E1;EPRS;EXOSC2;EXOSC3;EXOSC7;EXOSC8;EXOSC9;FARSA;FARSB;FTSJ1;GARS;GRSF1;GTF3C2;GTF3C6;HSD17B10;IARS;IARS2;KARS;LAGE3;LSM6;MARS;MARS2;METTL1;METTL2A;METTL2B;METTL6;MOCS3;NARS;NAT10;NSUN2;POLR3K;POP1;POP4;POP7;PPA1;PPA2;PUS1;PUS7;QRSL1;RARS;RPP21;RPP25;RPP30;RPP38;RPP40;SEPHS1;SEPHS2;SSB;TARBP1;TARS;TARS2;THUMPD2;THUMPD3;TP53RK;TPRKB;TRIT1;TRMT12;TRMT6;TRNT1;TSEN15;TSEN54;USP14;VARS;WARS;WDR4;YARS;YARS2;ZBTB8OS |
| rRNA metabolic process | | ABT1;BMS1;BOP1;BYSL;C1D;DCAF13;DDX21;DDX27;DDX47;DDX49;DDX52;DDX56;DKC1;EBNA1BP2;EIF4A3;EMG1;ERI3;ESF1;EXOSC2;EXOSC3;EXOSC4;EXOSC5;EXOSC6;EXOSC7;EXOSC8;EXOSC9;FBL;FTSJ3;GAR1;GTF2H5;GTF3A;GTF3C2;GTF3C6;GTPBP4;H2AFY;HEATR1;IMP4;ISG20L2;LAS1L;LSM6;LYAR;MARS;MPHOSPH10;MPHOSPH6;MRPL1;MRPS11;MRPS9;MRTO4;NAT10;NCL;NGDN;NHP2;NOC4L;NOL10;NOL11;NOLC1;NOP10;NOP14;NOP2;NOP56;NOP58;NPM3;NSUN5;NVL;PA2G4;PDCD11;PELO;PES1;POLR1B;POP4;POP5;PWP1;RCL1;RIOK1;RPF2;RPL7;RPL7L1;RPS21;RPS27;RRP1;RRP15;RRP1B;RRP9;RRS1;RSL1D1;SIRT7;SUV39H1;TEX10;TFB2M;TSR1;UTP14A;UTP15;UTP18;UTP20;UTP23;UTP6;WDR12;WDR3;WDR43;WDR46;WDR74;WDR75;ZNHIT3 |
| translational elongation | | AARS;CHCHD1;DAP3;DPH2;DPH3;EIF4A3;EIF5A;EIF5AL1;ERAL1;GFM1;HBS1L;MRPL1;MRPL11;MRPL12;MRPL13;MRPL14;MRPL15;MRPL17;MRPL18;MRPL19;MRPL2;MRPL21;MRPL22;MRPL24;MRPL27;MRPL3;MRPL32;MRPL33;MRPL35;MRPL36;MRPL37;MRPL38;MRPL39;MRPL4;MRPL42;MRPL44;MRPL46;MRPL47;MRPL48;MRPL50;MRPL51;MRPL52;MRPL55;MRPL9;MRPS10;MRPS11;MRPS12;MRPS14;MRPS15;MRPS16;MRPS17;MRPS18A;MRPS18B;MRPS18C;MRPS2;MRPS21;MRPS22;MRPS23;MRPS24;MRPS25;MRPS28;MRPS33;MRPS35;MRPS5;MRPS6;MRPS7;MRPS9;SRP9;TSFM |

Abbreviations: LeadingEdgeNum, the number of leadingedge genes; FDR, false discovery rate from Benjamini and Hochberg from gene set enrichment analysis (GSEA).
