# Supplementary material for: A TIMM17A Regulatory Network Contributing to Breast Cancer
Source: Front Genet. 2021 Aug 5;12:658154. doi: 10.3389/fgene.2021.658154 (PMC8375323; doi:10.3389/fgene.2021.658154)
Supplement: Supplementary Table 3 — Significantly enriched GO annotations (molecular functions) of TIMM17A in breast carcinoma (LinkedOmics). [file Table_3.DOCX]

**Supplementary Table 3. Significantly enriched GO annotations (molecular functions) of TIMM17A in breast carcinoma (LinkedOmics).**

| description | leadingEdgeNum | FDR | LeadingEdgeGene |  |
| --- | --- | --- | --- | --- |
| structural constituent of ribosome | 59 | 0 | DAP3;MRPL1;MRPL11;MRPL12;MRPL13;MRPL14;MRPL15;MRPL17;MRPL18;MRPL19;MRPL2;MRPL21;MRPL22;MRPL24;MRPL27;MRPL3;MRPL32;MRPL33;MRPL35;MRPL36;MRPL37;MRPL4;MRPL42;MRPL46;MRPL47;MRPL51;MRPL52;MRPL55;MRPL9;MRPS11;MRPS12;MRPS14;MRPS15;MRPS16;MRPS17;MRPS18A;MRPS18B;MRPS18C;MRPS2;MRPS21;MRPS22;MRPS23;MRPS24;MRPS25;MRPS33;MRPS35;MRPS5;MRPS6;MRPS7;MRPS9;NDUFA7;RPL22L1;RPL26L1;RPL30;RPL38;RPL39;RPL39L;RPL7;RPL7L1 |  |
| unfolded protein binding | 40 | 0 | AFG3L2;CALR;CCT2;CCT3;CCT4;CCT5;CCT6A;CCT7;CCT8;CHAF1A;CHAF1B;DNAJA1;DNAJA3;DNAJB11;GRPEL1;GRPEL2;HEATR3;HSP90AA1;HSP90AB1;HSP90B1;HSPA14;HSPA5;HSPA8;HSPA9;HSPD1;HSPE1;MKKS;NPM1;PFDN2;PFDN4;PFDN6;PPIA;PPIAL4C;PPIAL4G;PPID;PPIH;PTGES3;TCP1;TOMM20;TRAP1 | |
| catalytic activity, acting on RNA | 107 | 0 | AARS;CARS;CPSF3;CRCP;DARS2;DBR1;DDX1;DDX10;DDX18;DDX56;DHX57;DHX9;DQX1;EIF4A1;EIF4A3;EMG1;EPRS;ERI3;EXO1;EXOSC2;EXOSC3;EXOSC4;EXOSC5;EXOSC6;EXOSC7;EXOSC9;FARSA;FARSB;FBL;FEN1;FTSJ1;FTSJ3;G3BP1;GARS;IARS;IARS2;ISG20L2;KARS;LACTB2;MARS;MARS2;MED20;METTL1;METTL2A;METTL2B;METTL6;MRPL44;NARS;NOP2;NSUN2;PIF1;PNPT1;POLR1B;POLR1C;POLR2B;POLR2C;POLR2D;POLR2F;POLR2G;POLR2H;POLR2K;POLR3C;POLR3F;POLR3G;POLR3K;POP1;POP4;POP7;PRIM1;PRIM2;PRPF18;PTRH2;PUS1;QRSL1;RAD54B;RARS;RNASEH1;RNASEH2A;RPP21;RPP25;RPP30;RPP38;RPP40;SMG5;SMG7;SUPV3L1;TARBP1;TARS;TARS2;TDP2;TERT;TFB2M;TGS1;THUMPD2;THUMPD3;TRIT1;TRMT12;TRNT1;TSEN15;TSEN54;TSN;TSNAX;VARS;WARS;WDR4;YARS;YARS2 | |
| single-stranded DNA binding | 40 | 0 | APTX;BLM;CDC45;DHX9;GEN1;HMGB2;HNRNPA2B1;HNRNPU;HSF1;HSPD1;LRPPRC;MCM10;MCM4;MCM6;MCM7;MSH2;NEIL3;NME1;NUCKS1;NUP35;POLR2D;POLR2G;POLR2H;POLR3C;PRIM1;PRIM2;RAD18;RAD23B;RAD51;RAD51AP1;RECQL4;RPA3;SMC2;SMC4;SSBP1;TDP2;TERF1;TSN;TSNAX;YBX1 | |
| electron transfer activity | 52 | 0 | CIAPIN1;COX10;COX11;COX4I1;COX5A;COX5B;COX6A1;COX6B1;COX6C;COX7A2;COX7A2L;COX7B;COX7C;COX8A;CYC1;CYCS;DEGS1;DHDH;DLD;GLRX2;GLRX3;GLRX5;IDH3B;IDO1;ME1;ME2;NDUFA12;NDUFA4;NDUFAF2;NDUFS1;NDUFS2;NDUFS3;NDUFS6;NDUFV2;NQO1;P4HA2;PHGDH;RDH16;SDHA;SDHB;SDHC;SRD5A1;TSTA3;TXNRD1;UQCR10;UQCR11;UQCRB;UQCRC1;UQCRFS1;UQCRH;UQCRHL;UQCRQ | |

Abbreviations: LeadingEdgeNum, the number of leadingedge genes; FDR, false discovery rate from Benjamini and Hochberg from gene set enrichment analysis (GSEA).
