# Supplementary material for: A TIMM17A Regulatory Network Contributing to Breast Cancer
Source: Front Genet. 2021 Aug 5;12:658154. doi: 10.3389/fgene.2021.658154 (PMC8375323; doi:10.3389/fgene.2021.658154)
Supplement: Supplementary Table 4 — Significantly enriched KEGG pathway annotations of TIMM17A in breast carcinoma (LinkedOmics). [file Table_4.DOCX]

**Supplementary Table 4. Significantly enriched KEGG pathway annotations of TIMM17A in breast carcinoma (LinkedOmics).**

| description | leadingEdgeNum | FDR | LeadingEdgeGene |  |
| --- | --- | --- | --- | --- |
| Proteasome | 34 | 0 | ADRM1;POMP;PSMA1;PSMA2;PSMA3;PSMA4;PSMA5;PSMA6;PSMA7;PSMB1;PSMB2;PSMB3;PSMB4;PSMB5;PSMB6;PSMB7;PSMC1;PSMC2;PSMC3;PSMC4;PSMC5;PSMC6;PSMD1;PSMD11;PSMD12;PSMD13;PSMD14;PSMD2;PSMD3;PSMD4;PSMD7;PSMD8;PSME3;PSME4 |  |
| Spliceosome | 53 | 0 | BCAS2;BUD31;CDC5L;CTNNBL1;EFTUD2;EIF4A3;HNRNPA3;HNRNPC;HNRNPU;HSPA1B;HSPA8;LSM2;LSM3;LSM4;LSM5;LSM6;MAGOH;MAGOHB;NCBP1;NCBP2;PHF5A;PPIH;PPIL1;PRPF18;PRPF3;PRPF4;PRPF40A;PUF60;RBM17;RBM8A;SF3A3;SF3B3;SF3B4;SF3B5;SMNDC1;SNRNP40;SNRPA;SNRPA1;SNRPB;SNRPB2;SNRPC;SNRPD1;SNRPD2;SNRPD3;SNRPE;SNRPF;SNRPG;THOC3;TRA2B;TXNL4A;U2AF1;USP39;ZMAT2 | |
| Ribosome | 42 | 0 | MRPL1;MRPL11;MRPL12;MRPL13;MRPL14;MRPL15;MRPL17;MRPL18;MRPL19;MRPL2;MRPL21;MRPL22;MRPL24;MRPL27;MRPL3;MRPL32;MRPL33;MRPL35;MRPL36;MRPL4;MRPL9;MRPS10;MRPS11;MRPS12;MRPS14;MRPS15;MRPS16;MRPS17;MRPS18A;MRPS18C;MRPS2;MRPS21;MRPS5;MRPS6;MRPS7;MRPS9;RPL22L1;RPL26L1;RPL30;RPL38;RPL39;RPL7 | |
| Oxidative phosphorylation | 62 | 0 | ATP6V0A4;ATP6V0B;ATP6V0E1;ATP6V1C1;ATP6V1C2;ATP6V1E1;ATP6V1F;ATP6V1H;COX10;COX11;COX17;COX4I1;COX5A;COX5B;COX6A1;COX6B1;COX6C;COX7A2;COX7A2L;COX7B;COX7C;COX8A;CYC1;NDUFA1;NDUFA11;NDUFA12;NDUFA4;NDUFA6;NDUFA7;NDUFA8;NDUFA9;NDUFAB1;NDUFB10;NDUFB11;NDUFB2;NDUFB3;NDUFB4;NDUFB5;NDUFB6;NDUFB9;NDUFC1;NDUFS1;NDUFS2;NDUFS3;NDUFS4;NDUFS5;NDUFS6;NDUFV2;NDUFV3;PPA1;PPA2;SDHA;SDHB;SDHC;UQCR10;UQCR11;UQCRB;UQCRC1;UQCRFS1;UQCRH;UQCRHL;UQCRQ | |
| electron transfer activity | 38 | 0 | BMS1;CSNK2B;DKC1;EIF6;EMG1;GAR1;GNL2;GNL3;GTPBP4;HEATR1;IMP4;LSG1;MPHOSPH10;NAT10;NHP2;NOP10;NOP56;NOP58;NVL;NXT1;POP1;POP4;POP7;PWP2;RAN;RBM28;RIOK1;RPP25;RPP38;RPP40;TCOF1;UTP14A;UTP18;UTP6;WDR3;WDR43;WDR75;XPO1 | |

Abbreviations: LeadingEdgeNum, the number of leading edge genes; FDR, false discovery rate from Benjamini and Hochberg from gene set enrichment analysis (GSEA).
