# Supplementary material for: A TIMM17A Regulatory Network Contributing to Breast Cancer
Source: Front Genet. 2021 Aug 5;12:658154. doi: 10.3389/fgene.2021.658154 (PMC8375323; doi:10.3389/fgene.2021.658154)
Supplement: Supplementary Table 5 — Significantly enriched kinase-target networks of TIMM17A in breast carcinoma (LinkedOmics). [file Table_5.DOCX]

**Supplementary Table 5. Significantly enriched kinase-target networks of *TIMM17A* in breast carcinoma (LinkedOmics).**

| **Geneset** | **LeadingEdgeGene** |
| --- | --- |
| Kinase_CDK1 | ANAPC11;BIRC5;BLM;BRCA1;BUB1;BUB1B;CARHSP1;CCNB1;CDC20;CDC25A;CDC25B;CDC25C;CDC7;CDCA5;CENPA;CEP55;CHEK1;CKAP2;CSNK2B;DLGAP5;DNM1L;DTL;DUT;E2F1;ECT2;EIF4EBP1;EIF4G1;ELAVL1;ERCC6L;ESPL1;EZH2;FANCG;FEN1;FOXK2;FOXM1;GMPS;HMGA1;HMGCS1;KIF11;KIF20B;KIF22;KIF2C;LBR;LDHA;LMNB1;LMNB2;MCM7;MCTS1;MKI67;NCAPG;NCL;NME1;NME2;NOLC1;NPM1;NUCKS1;NUSAP1;PAICS;PBK;PDIA3;PI4KB;PRC1;PRDX1;PYCR2;RAB4A;RCC1;RFC2;RFC3;RFC4;RFC5;RRM2;SLBP;SPAG5;SSR1;STMN1;TCOF1;TERF1;TK1;TMPO;TOP2A;TPR;TPX2;UBAP2L;UBE2A;UBE2I;UHRF1;UNG;USP1;USP14;XPO1;ZC3H11A |
| Kinase_PLK1 | ANAPC7;BIRC5;BUB1B;CCNB1;CDC25A;CDC25B;CDC25C;CDC6;CENPQ;CEP55;CHEK2;CLSPN;ERCC6L;ESPL1;FBXO5;FOXM1;GTSE1;HNRNPU;KIF2C;NPM1;PKMYT1;PRC1;RACGAP1;RAD51;RAN;RUVBL1;STIL;TERF1;TOP2A |
| Kinase_AURKB | AURKB;BIRC5;CCDC86;CDCA2;CDCA5;CDCA8;CENPA;CHMP4C;CKAP2;DDX52;DEK;DSN1;H3F3A;HIST1H3B;HIST2H3C;HSP90AB1;INCENP;KIF23;KIF2C;KIF4A;MKI67;MPHOSPH10;NDC80;NSUN2;NUSAP1;PLK1;PRKDC;RACGAP1;RBM14;SHCBP1;SUPT16H |
| Kinase_CDK2 | AKIRIN2;ANAPC10;ANAPC11;ANAPC7;BLM;BRCA1;C1orf198;C9orf40;CCNA2;CCNE1;CDC20;CDC25C;CDC5L;CDC6;CDC7;CDK16;CDK2;CDT1;CENPF;CHEK1;CRTC2;CSNK2B;DIAPH3;DLGAP5;DNM1L;DTL;E2F1;E2F2;E2F3;EIF4A3;EIF4G1;ELAVL1;ERAL1;EZH2;FOXK2;FOXM1;GMPS;GORASP2;HMGA1;KIF22;LARP1;LMNB2;MCM2;MCM3;MCM4;MCM7;MKI67;MRPS18B;MTHFD1L;MYBL2;NBN;NCAPH;NCL;NPM1;NUCKS1;NUP107;NUP133;PAICS;PRPF3;PYCR1;PYCR2;RAD18;RBL1;RRM2;SETDB1;SKP2;SNAPIN;STMN1;TBCE;TCOF1;TK1;TOPBP1;TPR;TPX2;TSN;TSR1;TUBG1;UBE2A;UBE2O;UHRF1;UNG;XRCC6;ZC3H11A |
| Kinase_CHEK1 | AURKB;BLM;CDC25A;CDC25B;CDC25C;CDK1;CHEK1;CLSPN;CSNK2B;E2F3;E2F6;EBNA1BP2;EPRS;FANCD2;FANCE;FEN1;H3F3A;HNRNPA2B1;IDI1;KIFC1;KPNA2;MAD2L1;MCM3;MKI67;POP4;PPP2R5D;PRPF3;RACGAP1;RAD51;RBM14;RRP12;SSBP1;TLK2;XRCC6;YBX1;YBX2 |

Abbreviations: LeadingEdgeNum, the number of leading edge genes; FDR, false discovery rate from Benjamini and Hochberg from gene set enrichment analysis (GSEA).
