# Supplementary material for: A TIMM17A Regulatory Network Contributing to Breast Cancer
Source: Front Genet. 2021 Aug 5;12:658154. doi: 10.3389/fgene.2021.658154 (PMC8375323; doi:10.3389/fgene.2021.658154)
Supplement: Supplementary Table 6 — Significantly enriched miRNA-target networks of TIMM17A in breast carcinoma (LinkedOmics). [file Table_6.DOCX]

**Supplementary Table 6. Significantly enriched miRNA-target networks of *TIMM17A* in breast carcinoma (LinkedOmics).**

| **Geneset** | **LeadingEdgeGene** |
| --- | --- |
| CCAGGGG,MIR-331 | ALX4;ATOH8;CRIM1;DNAJB5;EGR3;FAM160B2;GGT7;GRASP;HOXA3;KHNYN;KSR1;LDLRAD2;LRCH4;MBD6;MEIS1;MEIS2;MINK1;NBEA;PHC2;PRDM8;SEMA6A;SPRY4;TAOK2;TSPAN18;YPEL4;ZNF513;ZNF609 |
| GAGCCTG,MIR-484 | ACVR1B;CBL;DACH1;DENND5A;DLEC1;DLL4;DPYSL2;EDA;EZH1;FAM13A;FGF1;FOXO4;GOLGA6B;HIPK1;HOXA5;HSPG2;KDM4A;KLF12;LBH;MINK1;MYCBP2;NFIA;NFKBIZ;PCDH19;PCDH7;PHC2;PLEKHH2;PRKCB;PTGER4;PTPRE;SCARA3;SORBS2;TAF1L;TRIOBP;ZFYVE1;ZNF667 |
| GACAATC,MIR-219 | AFAP1L2;AFF4;AKAP13;APPL2;ARID3B;BTBD7;CBFA2T3;CELF2;CGNL1;CPEB2;CPEB3;CSRNP3;CXXC5;EGR3;EPHA4;ERG;ESR1;ETV5;FAM160A2;FBXL17;FZD4;GBA2;GTPBP1;HAS3;ING3;INPP5J;ITSN1;KCNH8;KCNJ2;KLF9;LCOR;MAPT;MEF2D;MFNG;MKNK2;NCOA1;NR2C2;NRIP1;PDGFRA;PDZRN4;PI4K2A;PIP5K1C;PODXL;PPARGC1A;PURG;RBM24;RECK;SCN5A;SH3D19;SLC30A4;SLK;SNRK;SP4;SYNGAP1;TACC1;TGFBR2;THRB;TRHDE;UBR1;ZC3H12B;ZCCHC24;ZDHHC2;ZNF609;ZNF827 |
| CCCAGAG,MIR-326 | ADAMTS6;AHCYL2;ANKFY1;ATP8B2;ATXN1;CDH22;CELF2;CIC;CORO2B;CRIM1;DLGAP2;DRD2;EML2;FNDC3A;GGT7;GNAO1;H6PD;ITSN1;KCNIP2;KLHL14;LRRC32;NAV3;NHS;NRP1;OGT;PALM;PURG;RPGR;RPS6KA1;RPS6KA3;SCARF1;SEMA6D;SMAD6;ST3GAL3;SYNGAP1;TCF4;TLN1;UBXN10;VPS39;ZMIZ1;ZNF609 |
| AGCTCCT,MIR-28 | AMIGO1;ARID1A;ATXN1;BCAM;CLK3;CRIM1;CSF1;FOXN3;GPM6A;IKZF4;IQSEC2;KIAA0355;LBH;LUZP1;MAP3K3;NNAT;RCVRN;SMAD3;TLN2;TMEM200B;TNFSF12;VAMP2;YPEL3;ZC3H12B |

Abbreviations: LeadingEdgeNum, the number of leading edge genes; FDR, false discovery rate from Benjamini and Hochberg from gene set enrichment analysis (GSEA).
