# Supplementary material for: A TIMM17A Regulatory Network Contributing to Breast Cancer
Source: Front Genet. 2021 Aug 5;12:658154. doi: 10.3389/fgene.2021.658154 (PMC8375323; doi:10.3389/fgene.2021.658154)
Supplement: Supplementary Table 7 — Significantly enriched transcription factor-target networks of TIMM17A in breast carcinoma (LinkedOmics). [file Table_7.DOCX]

**Supplementary Table 7. Significantly enriched transcription factor-target networks of *TIMM17A* in breast carcinoma (LinkedOmics).**

| **Geneset** | **LeadingEdgeGene** |
| --- | --- |
| GGAANCGGAANY_UNKNOWN | ATP6V1E1;BANF1;BMS1;COX6B1;COX7A2;CSNK2B;DDX55;DPM1;EBNA1BP2;EIF1AD;EIF2S1;EIF3H;FARSA;GLRX5;HSPH1;MED8;MOCS3;MRPL21;MRPS18A;MRPS21;MRPS23;NCBP2;POMP;PRPF3;PSMB4;RARS;RPL38;RUVBL2;SDF2;SEC61A1;SEC61G;SLC25A44;SNRPE;SRP54;TFB2M;THUMPD3;TIMM8A;TMCO1;UBL5;UGGT1 |
| V$E2F_Q6 | ACBD6;AK2;APH1A;ARHGAP11A;ATAD2;ATAD5;CDC25A;CDC45;CDC6;CDCA7;CDK1;CDT1;CLSPN;DCTPP1;DNAJC9;E2F1;E2F3;E2F7;E2F8;EZH2;FANCD2;FBXO5;GAPDH;GEN1;GINS3;GMNN;GSPT1;H2AFZ;HIST1H2BK;INTS7;JPH1;KPNB1;MCM2;MCM3;MCM4;MCM6;MCM7;MCM8;MSH2;MXD3;NCL;NOLC1;PCNA;PHF5A;PKMYT1;POLA2;POLE2;PPP1CC;PRKDC;RAD51;RANBP1;RRM2;SNRPD1;STMN1;SUMO1;TMPO;TOPBP1;TRA2B;TRMT6;UNG;WDR62;YBX2;ZNF367 |
| V$E2F1_Q6 | ACBD6;AK2;AP1S1;APH1A;ARHGAP11A;ATAD2;ATAD5;CAND1;CBX3;CDC25A;CDC5L;CDC6;CDCA7;CDK1;CDT1;CLSPN;DCK;DCTPP1;DNAJC9;E2F1;E2F3;E2F7;E2F8;EED;EZH2;FANCC;FANCD2;FANCG;FBXO5;GAPDH;GEN1;GINS3;GMNN;GPN3;GSPT1;H2AFZ;HIST1H2AH;HIST1H2BK;HMGA1;HNRNPA2B1;HNRNPD;HOXC10;KPNB1;MCM2;MCM3;MCM4;MCM6;MCM7;MCM8;MSH2;MXD3;NASP;NCL;NOLC1;NUFIP2;PAQR4;PCNA;PHF5A;PKMYT1;POLE2;POLE4;PRKDC;PRPS2;RANBP1;RBL1;RRM2;SASS6;SERBP1;SLC25A3;SMC6;SNRPD1;STMN1;SUMO1;SUV39H1;SYNCRIP;SYNGR4;TMPO;TOPBP1;TRA2B;TRMT6;TYRO3;UGGT1;UNG;WDR62;YBX2;ZBTB8OS;ZNF367 |
| SGCGSSAAA_V$E2F1DP2_01 | ACBD6;AK2;AP1S1;ATAD2;ATAD5;CAND1;CDC25A;CDC6;CDCA7;CLSPN;DCK;DCTPP1;DNAJC9;E2F1;E2F3;E2F8;EED;FANCC;FANCG;FBXO5;GEN1;GINS3;GMNN;H2AFZ;HIST1H2AH;HIST1H2BK;HMGA1;HNRNPD;HOXC10;MCM2;MCM3;MCM4;MCM6;MCM7;MSH2;MXD3;NASP;NCL;NOLC1;PAQR4;PCNA;PHF5A;PKMYT1;POLE2;POLE4;PRKDC;PTMA;RANBP1;RRM2;SMC6;SNRPD1;SUV39H1;SYNCRIP;SYNGR4;TOPBP1;TRA2B;TYRO3;UGGT1;UNG;WDR62;YBX2;ZNF367 |
| V$E2F_02 | ACBD6;AK2;AP1S1;APH1A;ARHGAP11A;ATAD2;ATAD5;CAND1;CBX3;CDC25A;CDC5L;CDC6;CDCA7;CDK1;CLSPN;DCK;DCTPP1;DNAJC9;E2F1;E2F3;E2F7;E2F8;EED;EIF4A1;EZH2;FANCC;FANCD2;FANCG;FBXO5;GAPDH;GEN1;GINS3;GMNN;GSPT1;H2AFZ;HIST1H2AH;HIST1H2BK;HMGA1;HNRNPA2B1;HNRNPD;HOXC10;MCM2;MCM3;MCM4;MCM6;MCM7;MCM8;MSH2;MXD3;NASP;NCL;NOLC1;NUFIP2;PAQR4;PCNA;PHF5A;PKMYT1;POLE2;POLE4;PRKDC;PRPS2;PTMA;RANBP1;RBL1;RRM2;SASS6;SMC6;SNRPD1;STMN1;SUMO1;SUV39H1;SYNCRIP;SYNGR4;TMPO;TOPBP1;TRA2B;TRMT6;TYRO3;UGGT1;UNG;WDR62;YBX2;ZNF367 |

Abbreviations: LeadingEdgeNum, the number of leading edge genes; FDR, false discovery rate from Benjamini and Hochberg from gene set enrichment analysis (GSEA). V$, the annotation found in Molecular Signatures Database (MSigDB) for tran-scription factors (TF).
