# Supplementary material for: A TIMM17A Regulatory Network Contributing to Breast Cancer
Source: Front Genet. 2021 Aug 5;12:658154. doi: 10.3389/fgene.2021.658154 (PMC8375323; doi:10.3389/fgene.2021.658154)
Supplement: Supplementary Table 8 — The sequences of primers used for qPCR. [file Table_8.docx]

**Supplementary Table S8. The sequences of primers used for qPCR.**

| **Gene** | **Sense Primer** | **Antisense Primer** |
| --- | --- | --- |
| TIMM17A | 5’-ACGAGGGAGTTTGACAGCTA -3’ | 5’-CCATTTCTTGCTGCCAGTAT-3’ |
| CDK1 | 5’- TGGAAGCTAGGGTAGTCTGG -3’ | 5’-TGGTTTGGTAGAACTGGTGC -3’ |
| 18s | 5’- CCTGGATACCGCAGCTAGGA-3’ | 5’- GCGGCGCAATACGAATGCCCC -3’ |
