# Supplementary material for: A TIMM17A Regulatory Network Contributing to Breast Cancer
Source: Front Genet. 2021 Aug 5;12:658154. doi: 10.3389/fgene.2021.658154 (PMC8375323; doi:10.3389/fgene.2021.658154)
Supplement: Supplementary file 12 [file Data_Sheet_1.docx]

**Legends**

**Figure 1 – *TIMM17A* transcription in breast carcinoma (Oncomine^TM^).**

Levels of *TIMM17A* mRNA and DNA copy number were significantly higher in breast carcinoma than in normal tissue. Shown are fold change, associated p values, and overexpression rank based on Oncomine^TM^ analysis. (A–D) Box plots showing *TIMM17A* mRNA levels for The Cancer Genome Atlas (TCGA) Invasive Ductal Breast Carcinoma, TCGA Invasive Breast Carcinoma, Curtis Invasive Ductal Breast Carcinoma, and Curtis Breast Carcinoma datasets, respectively. (E–F) Box plots showing *TIMM17A* copy number in TCGA Breast 2 and Curtis Breast 2 datasets, respectively.

**Figure 2 – Kaplan‒Meier survival curves showing significantly reduced overall survival (OS) and shorter distant metastases-free survival (DMFS) among breast cancer patients with high levels of *TIMM17A* expression.**

Clinical survival outcomes of breast cancer patients with high or low expression levels of TIMM17A. (A) OS in TCGA cohorts. (B) DMFS in TCGA cohorts. (C) OS in the GSE45255 cohort. (D) DMFS in the GSE45255 cohort. (E) OS in the GSE7390 cohort. (F) DMFS in the GSE7390 cohort. Plots are truncated at 10 years.

**Figure 3 – *TIMM17A* transcription in breast cancer (BRCA) patient subgroups stratified by sex, age, ethnicity, cancer stage, and metastasis (UALCAN).**

(A) Boxplot showing relative expression of *TIMM17A* in normal individuals of either sex and separately for male and female BRCA patients. (B) Boxplot showing relative expression of *TIMM17A* in normal individuals of any age and separately for BRCA patients aged 21–40, 41–60, 61–80, or 81–100 years. (C) Boxplot showing relative expression of TIMM17A in normal individuals of any ethnicity and separately for BRCA patients of Caucasian, African-American, or Asian ethnicity. (D) Boxplot showing relative expression of *TIMM17A* in normal individuals and separately for BRCA patients in stages 1, 2, 3, or 4. (E) Boxplot showing relative expression of *TIMM17A* in normal individuals and separately for BRCA patients with regional lymph node metastasis N0, N1, N2, or N3 tumors. (F) Boxplot showing relative expression of *TIMM17A* in normal individuals and separately for BRCA patients with regional lymph node metastasis N0, N1, N2 or N3 tumors. Data are mean ± SE. *, P < 0.05; **, P < 0.01; ***, P < 0.001.

**Figure 4 - Visual summary of TIMM17A alterations and biological interaction networks in breast carcinoma (cBioPortal).**

(A) OncoPrint of *TIMM17A* alterations in BRCA. The OncoPrint provides an overview of genomic alterations in *TIMM17A* affecting individual samples (columns) in BRCA from the TCGA dataset. The different types of genetic alterations are indicated by unique colors. (B) Network view of the *TIMM17A* neighborhood in BRCA. *TIMM17A* is the seed gene (indicated with thick border), and all other genes altered in BRCA are automatically identified Darker red indicates increased alteration frequency in BRCA. The interaction types are derived from the Biological Pathway Ex-change (BioPAX). The red connection indicates proteins are members of the same complex.

**Figure 5 - Enrichment analysis of TIMM17A neighbor genes altered in breast carcinoma.**

The bubble diagrams display the enrichment results of the top 50 *TIMM17A* neighbor genes altered in BRCA. (A) Disease Ontology (DO). (B) Gene ontology (GO). (C) Kyoto Encyclopedia of Genes and Genomes (KEGG). (D) Reactome analysis.

**Figure 6 - Genes differentially expressed in correlation with TIMM17A expression among breast carcinoma patients (LinkedOmics).**

(A) Pearson’s correlation analyses to assess the association between *TIMM17A* expression and that other genes differentially expressed in BRCA. (B–C) Heat maps showing genes positively and negatively correlated with *TIMM17A* expression in BRCA (TOP 50). Red indicates positively correlated genes and green indicates negatively correlated genes.

**Figure 7 - Significantly enriched GO annotations and KEGG pathways of *TIMM17A* network genes in breast carcinoma.**

GO annotations and KEGG pathways of genes with expression levels associated with TIMM17A expression in BRCA analyzed using GSEA. (A) Cellular components. (B) Biological processes. (C) Molecular functions. (D) KEGG pathway analysis. The blue column represents −log10(p). (E) KEGG pathway annotations of the cell cycle pathway. Red marked nodes are associated with the LeadingEdgeGene.

**Figure 8 - Cell cycle analysis of BRCA cells**

(A) Cell cycle analysis of BT-549 cells by flow cytometry (B) Cell cycle analysis of TIMM17A knockdown BT-549 cells by flow cytometry (C) Cell cycle analysis of SK-BR-3 cells by flow cytometry (D) Cell cycle analysis of TIMM17A knockdown SK-BR-3 cells by flow cytometry (E) Cell cycle distribution of siRNA-SK-BR-3, NC-SK-BR-3, siRNA-BT-549 and NC-BT-549.

**Figure 9 - Protein‒protein interaction network of ATR kinase targets (GeneMANIA).**

Protein‒protein interaction (PPI) network and functional analysis of the gene set enriched in targets of CDK1. Different colors of the network edge indicate the bioinformatics methods applied: co-expression, co-localization, physical interactions, pathway, predicted, genetic interactions, and shared protein domains. The different colors for the network nodes indicate the biological functions of the set of enrichment genes.

**Figure 10 – Associations of TIMM17A expression with CDK1 expression and microRNAs expression levels**

(A)Relationship between TIMM17A and CDK1 expression levels (B)Western blot analysis showing the effects of TIMM17A knock down on the expression levels of CDK1 and GAPDH (C) RNA electrophoretogram of miRNA 219, miRNA 326, and miRNA 331 in BRCA cells (D) Expression levels of miRNA 219, miRNA 326 and miRNA 331 in TIMM17A knockdown BRCA cells.

**Supplementary Figure 1- Correlations between TIMM17A expression and UBE2T,** **TMEM183A, SNRPE,** **CACYBP, and RABIF expression (LinkedOmics).**

The scatter plots show Pearson’s correlations between TIMM17A expression and the expression levels of UBE2T (A), TMEM183A (B), SNRPE (C), CACYBP (D), and RABIF (E).

**Supplementary Figure 2- Protein‒protein interaction network of miRNA 331 targets (GeneMANIA).**

Protein‒protein interaction (PPI) network and functional analysis of the gene set enriched in miRNA 331 targets. Different colors of the network edge indicate the bioinformatics method applied: co-localization, co-expression, shared protein domains, pathway, and genetic interactions. The different colors for the network nodes indicate the biological functions of the set of enrichment genes.

**Supplementary Figure 3- Protein‒protein interaction network of transcription factor E2F_Q6 targets (GeneMANIA).**

Protein‒protein interaction (PPI) network and functional analysis indicating the gene set enriched in transcription factor E2F_Q6 targets. Different colors of the network edge indicate the bioinformatics method applied: co-expression, physical interaction, pathway, co-localization, and predicted. The different colors for the network nodes indicate the biological functions of the set of enrichment genes.
